# Being Pathogenic, Plastic, and Sexual while Living with a Nearly Minimal Bacterial Genome

**DOI:** 10.1371/journal.pgen.0030075

**Published:** 2007-05-18

**Authors:** Pascal Sirand-Pugnet, Carole Lartigue, Marc Marenda, Daniel Jacob, Aurélien Barré, Valérie Barbe, Chantal Schenowitz, Sophie Mangenot, Arnaud Couloux, Beatrice Segurens, Antoine de Daruvar, Alain Blanchard, Christine Citti

**Affiliations:** 1 Université Bordeaux 2, UMR1090, Villenave d'Ornon, France; 2 INRA, UMR1090, Villenave d'Ornon, France; 3 INRA, ENVT, UMR1225, Toulouse, France; 4 Centre de Bioinformatique de Bordeaux, Université Bordeaux 2, Bordeaux, France; 5 Genoscope, Centre National de Séquençage, Evry, France; Université Paris V, INSERM U571, France

## Abstract

Mycoplasmas are commonly described as the simplest self-replicating organisms, whose evolution was mainly characterized by genome downsizing with a proposed evolutionary scenario similar to that of obligate intracellular bacteria such as insect endosymbionts. Thus far, analysis of mycoplasma genomes indicates a low level of horizontal gene transfer (HGT) implying that DNA acquisition is strongly limited in these minimal bacteria. In this study, the genome of the ruminant pathogen Mycoplasma agalactiae was sequenced. Comparative genomic data and phylogenetic tree reconstruction revealed that ∼18% of its small genome (877,438 bp) has undergone HGT with the phylogenetically distinct mycoides cluster, which is composed of significant ruminant pathogens. HGT involves genes often found as clusters, several of which encode lipoproteins that usually play an important role in mycoplasma–host interaction. A decayed form of a conjugative element also described in a member of the mycoides cluster was found in the M. agalactiae genome, suggesting that HGT may have occurred by mobilizing a related genetic element. The possibility of HGT events among other mycoplasmas was evaluated with the available sequenced genomes. Our data indicate marginal levels of HGT among *Mycoplasma* species except for those described above and, to a lesser extent, for those observed in between the two bird pathogens, M. gallisepticum and M. synoviae. This first description of large-scale HGT among mycoplasmas sharing the same ecological niche challenges the generally accepted evolutionary scenario in which gene loss is the main driving force of mycoplasma evolution. The latter clearly differs from that of other bacteria with small genomes, particularly obligate intracellular bacteria that are isolated within host cells. Consequently, mycoplasmas are not only able to subvert complex hosts but presumably have retained sexual competence, a trait that may prevent them from genome stasis and contribute to adaptation to new hosts.

## Author Summary

Mycoplasmas are cell wall–lacking prokaryotes that evolved from ancestors common to Gram-positive bacteria by way of massive losses of genetic material. With their minimal genome, mycoplasmas are considered to be the simplest free-living organisms, yet several species are successful pathogens of man and animal. In this study, we challenged the commonly accepted view in which mycoplasma evolution is driven only by genome down-sizing. Indeed, we showed that a significant amount of genes underwent horizontal transfer among different mycoplasma species that share the same ruminant hosts. In these species, the occurrence of a genetic element that can promote DNA transfer via cell-to-cell contact suggests that some mycoplasmas may have retained or acquired sexual competence. Transferred genes were found to encode proteins that are likely to be associated with mycoplasma–host interactions. Sharing genetic resources via horizontal gene transfer may provide mycoplasmas with a means for adapting to new niches or to new hosts and for avoiding irreversible genome erosion.

## Introduction

Organisms belonging to the *Mycoplasma* genus (class *Mollicutes*) are commonly described as the simplest and smallest self-replicating bacteria because of their total lack of cell wall, the paucity of their metabolic pathways, and the small size of their genome [1,2]. In the 1980s, they were shown to have evolved from more classical bacteria of the firmicutes taxon by a so-called regressive evolution that resulted in massive genome reduction [3,4].

One of the models attempting to improve understanding of the evolution of bacteria with small genomes proposes that erosion of bacterial genomes is more prone to occur in bacterial populations that are spatially isolated and sexually deficient [5]. In restricted habitats, the environment is rather steady and natural selection tends to be reduced, resulting in the inactivation of many genes by genetic drift [5,6]. In this scenario, DNA acquisition would be strongly limited, resulting, after losses of large genomic regions and accumulation of mutations, in genome stasis [7]. This evolution scheme is relevant for a number of obligate intracellular bacteria, including insect endosymbionts (e.g., *Buchnera* and Wigglesworthia spp.), and arguably for *Chlamydia*, and Rickettsia spp. The recent findings of a putative conjugative plasmid in Rickettsia felis [8] and of a substantial number of prophage, transposase and mobile-DNA genes in the insect endosymbiont Wolbachia pipientis challenged this model and it was proposed that gene inflow by horizontal gene transfer (HGT) may occur in some obligate intracellular species depending on their lifestyles [9].

**Figure 1:**
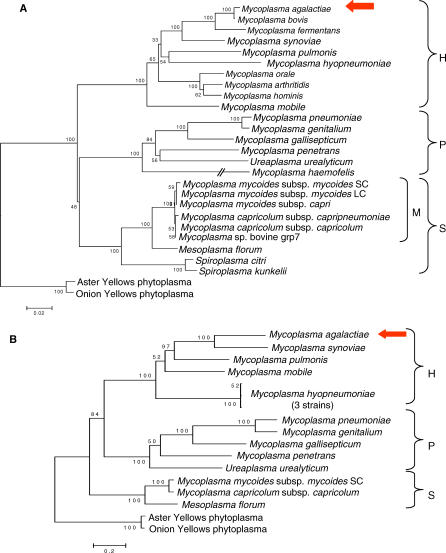
Phylogenetic Tree of Selected Mollicutes of the Spiroplasma/Mycoplasma Branch Inferred from 16S rDNA Sequences [3] or from Concatenated Shared Proteins (A) The tree was constructed using the distance (neighbor joining) method and the gaps complete deletion option of the MEGA2 software. A bootstrap of 500 replicates was performed; the number on each node indicates the percentage with which each branch topology was supported. The phylogenetic groups spiroplasma, pneumoniae, and hominis are indicated by S, P, and H, respectively. M, mycoides cluster. Candidatus *phytoplasma asteris* (Onion Yellows strain) and Aster Yellows phytoplasma were chosen as outgroup species. (B) 30 COGs shared by all sequenced mollicute genomes were extracted from the MolliGen database (see Materials and Methods). After alignment of each COG, the aligned sequences were concatenated. The tree was constructed using the maximum likelihood method (PhyML). A bootstrap of 100 replicates was performed; the number on each node indicates the percentage with which each branch topology was supported. The phylogenetic groups spiroplasma, pneumoniae, and hominis are indicated by S, P and H, respectively. Candidatus *phytoplasma asteris* (Onion Yellows strain) and Aster Yellows phytoplasma were chosen as outgroup species.

Mycoplasmas share with obligate intracellular bacteria a small genome size with marked AT nucleotide bias and a low number of genes involved in recombination and repair, but forces driving their evolution may not be quite the same, as they do have a very different lifestyle. Indeed, mycoplasmas mainly occur as extracellular parasites [10] and are often restricted to a living host, with some species having the ability to invade host cells [11]. They have a predilection for the mucosal surfaces of the respiratory and urogenital tracts, where they successfully compete for nutrients with many other organisms, establishing chronic infections (Table S1). Therefore, mycoplasma populations are far from being isolated and inhabit niches where exchange of genetic material may take place. The none-to-rare occurrence of HGT reported so far for mycoplasmas [12] is therefore surprising and seems to conflict with their lifestyle. On the other hand, HGT may depend on several other factors [9] that were described as limited or lacking in most mycoplasma species and that include an efficient machinery for recombination, genetic mobile elements such as prophages or conjugative plasmids, and a means for DNA uptake. However, this view of mycoplasma biology is changing, since homologous recombination has been demonstrated in these bacteria [13,14] and some new means of exchanging DNA are being discovered [15,16]. Indeed, several pathogenic mycoplasma species relevant to the veterinary field and the murine pathogen M. pulmonis were recently shown to form biofilms [17,18], structures that have been proposed to promote DNA exchange among bacteria. This finding, together with previous evidence for DNA transfer under laboratory conditions in M. pulmonis via conjugation [19], raises the exciting question of whether some mycoplasmas species are sexually competent. Subsequently, this would suggest that mycoplasma species which co-infect the same host niches might exchange genetic material. Remarkably, biofilm formation and the occurrence of an integrative conjugative element (ICE) have both been newly described in the M. agalactiae species [16,18]. This pathogen is responsible for contagious agalactia in small ruminants [20], a syndrome that includes mastitis, pneumonia, and arthritis and that is also caused by some members of the so-called mycoides cluster, such as M. capricolum subsp. *capricolum* and M. mycoides subsp. *mycoides* Large Colony. Although producing similar symptoms in the same host, these species belong to two distinct and distant branches of the mollicute phylogenetic tree (Figure 1). Their relative phylogenetic positions are irrespective of whether the tree is constructed from aligned 16S rDNA (Figure 1A) or from 30 aligned proteins shared by all living organisms [21] (Figure 1B). M. agalactiae belongs to the hominis phylogenetic branch, together with a closely related ruminant pathogen, M. bovis, while the six members that comprise the “mycoides cluster” belong to the spiroplasma phylogenetic branch [22]. Whole-genome sequences are available for two members of the mycoides cluster; M. mycoides subsp. *mycoides* SC [23], which is responsible for contagious bovine pleuropneumonia [24], and M. capricolum subsp. *capricolum* [25]. In contrast, there is a limited amount of sequence data available for M. agalactiae and M. bovis. Mycoplasmas that have been fully sequenced in the hominis phylogenetic group are a murine pathogen M. pulmonis [26], a swine pathogen M. hyopneumoniae (strain 232 [27]; strains 7748 and J [12]), an avian pathogen M. synoviae [12], and a mycoplasma isolated from fish, M. mobile [28] (Figure 1B).

Mechanisms underlying ruminant mycoplasma diseases have yet to be elucidated and very little is known regarding the mycoplasma factors that are involved in virulence and host interaction. Genes thus far identified in M. agalactiae and for which a function in relation to virulence has been predicted are (i) a family of phase-variable related surface proteins, designated as Vpma, which are encoded by a locus subjected to high-frequency DNA rearrangements and could be involved in adhesion [29,30], (ii) the P40 protein, which is involved in host–cell adhesion in vitro but is not expressed in all field isolates [31], and (iii) the P48 protein, which has homology to an M. fermentans product with a macrophage-stimulatory activity [32]. Several of these gene products have homologs in M. bovis but not in mycoplasmas of the mycoides cluster.

Whole-genome comparison between phylogenetically distant mycoplasmas that colonize the same host could provide a basis from which to comprehend the factors involved in mycoplasma host adaptation. With this initial goal, we sequenced the M. agalactiae genome of the pathogenic type strain PG2. Results revealed a classical mollicute genome with a coding capacity of 751 CDSs, half of which are annotated as encoding hypothetical products.

Unexpectedly, comparative analysis of the M. agalactiae genome with that of other mollicutes and bacteria suggests that a significant amount of genes (∼18 %) has been horizontally transferred to or acquired from mycoplasmas of the mycoides cluster that are phylogenetically distant while sharing common ruminant hosts. In light of these data, we re-examined mollicute genomes for HGT events with a particular focus on those that occurred after mycoplasmas branched into three phylogenetic groups (see Figure 1 for the hominis, pneumoniae, and spiroplasma phylogenetic groups). Our analyses confirm data so far reported regarding the low incidence of HGT between *Mycoplasma* species with the exception of that described in this study, between M. agalactiae and members of the mycoides cluster and, to a lesser extent, between M. gallisepticum and M. synoviae. To our knowledge, this is the first description of large-scale horizontal gene transfer between mycoplasmas.

## Results

### M. agalactiae: Overall Features of a Small Genome

The genome of the M. agalactiae type strain PG2 consists of a single, circular chromosome; general features are summarized in Table 1. The genome sequence was numbered clockwise starting from the first nucleotide of the *dnaA* gene, which was designated as the first CDS (MAG0010). This gene is involved in the early steps of the replication initiation process [33] and is typically located near mycoplasma origins of replication. Indeed, *dnaA* boxes flanking the *dnaA* gene were shown in M. agalactiae to promote free replication of the ColE1-based E. coli vectors in which they were cloned [34]. Although these experiments clearly localized the *M. agalactiae oriC* in the vicinity of the *dnaA* gene, whole-genome analysis did not indicate a significant GC-skew inversion [35] in this region (unpublished data). In contrast to other mycoplasma genomes [36], a high level of gene-strand bias was not observed, even when restricting the analysis to the *dnaA* vicinity.

**Table 1:**
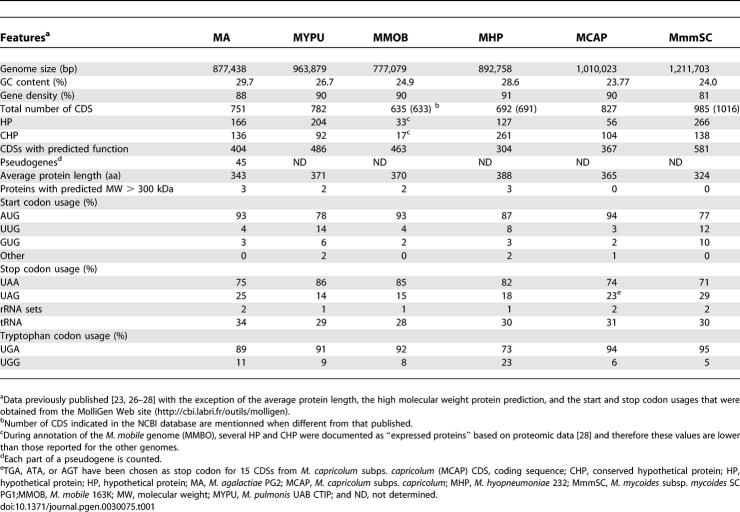
General Features of the M. agalactiae (MA) Genome Compared to Those of Mycoplasma Species of the Same Phylogenetic Group (MYPU, MMOB, MHP) and Other Phylogenetically Remote Ruminant Mycoplasmas (MCAP and MmmSC)

**Figure 2:**
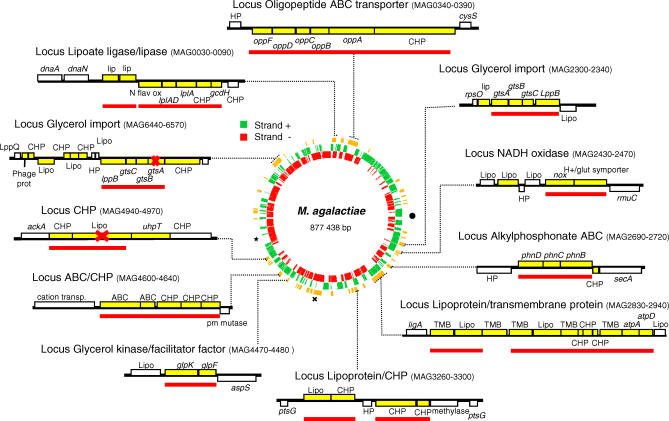
Schematic Presenting the Circular M. agalactiae Genome and the Location of Genes Inherited by Horizontal Transfer from the Mycoides Cluster The 136 genes potentially inherited from the mycoides cluster are shown on the outer circle as yellow bars, with the inner circles representing the positive (green) or the negative (red) strand. The two adjacent 16S-23S rRNA operons and the two 5S rRNA genes are represented by a star and a dot, respectively. The genomic organization of some transferred genes (yellow boxes above and below the line according to their relative orientation on the genome) is illustrated around the circular map, with pseudogenes indicated by a red cross and tRNAs by black arrowheads. Gene clusters presenting the same organization in M. agalactiae, M. mycoides subsp. *mycoides* SC, and M. capricolum subsp. *capricolum* are underlined by red bars. The ICE region, homologous to that of M. capricolum subsp. *capricolum* but missing in M. mycoides subsp. *mycoides* SC type strain, is noted by a cross. CHP, conserved hypothetical protein; HP, hypothetical protein; Lipo, predicted lipoprotein; and TMB, predicted transmembrane protein.

Overall, M. agalactiae strain PG2 possesses a typical mollicute genome, with a small size (877,438 bp), a low GC content (29.7 moles %), a high gene compaction (88% of coding sequence), and UGA preferentially used as a tryptophan codon over UGG (Table 1). Its GC% value is slightly higher than that observed for some other mycoplasma species but is close to the average GC content (28%) calculated from the 16 available mollicute genomes. Using the CAAT-box software package, 751 CDSs were identified, 404 (53.8%) of which had a predicted function. The genome also contains 34 tRNA genes and two nearly identical sets of rRNA genes with two 16S–23S rRNA operons (MAG16S1-MAG23S1 and MAG16S2-MAG;23S2) and the two 5S rRNA genes (MAG5S1 and MAG5S2) clustered in two loci separated from each other by ∼400 kb (Figure 2).

### HGT among Distant Mycoplasma Species Sharing the Same Host

Prediction of M. agalactiae CDS function was based on BLAST searches against SwissProt, trembl, and MolliGen databases. For CDSs showing significant similarities with database entries, most best BLAST hits (BBH) were found with M. synoviae and *M. pulmonis,* which belong, together with M. agalactiae, to the hominis phylogenetic group (Figure 1). Unexpectedly, a large number of BBH were also obtained with M. mycoides subsp. *mycoides* SC or M. capricolum subsp. *capricolum,* which both belong to the mycoides cluster (Figure S1). Since this cluster is exclusively composed of ruminant pathogens and is relatively distant from M. agalactiae in the mollicute phylogenetic tree (Figure 1), this prompted us to closely examine the corresponding CDSs. A total of 136 M. agalactiae CDSs were then identified as having their BBH with organisms from the mycoides cluster, with 50 having no significant similarity outside of this cluster (Table S2). Of the remaining 86, 73 also had a homolog in at least one in the four available genomes of the hominis group (*M. pulmonis, M. mobile, M. synoviae,* and M. hyopneumoniae) (Table S2) and 13 in other mollicutes or bacteria (Tables S2 and S3). Further phylogenetic tree reconstruction showed that 75 out of 86 CDSs display highly significant bootstrap values (≥ 90%) supporting HGT with homologs of the mycoides cluster. Among the 11 CDSs with low bootstrap values, six belong to gene clusters in which synteny is conserved in the mycoides cluster, three belong to an ICE element (see below) found in *M. agalactiae* and M. capricolum subsp. *capricolum* and two others were not further considered, suggesting that ∼134 CDS have undergone horizontal gene transfer in between mycoplasma(s) of the mycoides cluster and M. agalactiae or its ancestor.

Of the predicted transferred CDSs, nine and 22 have a homolog either in M. mycoides subsp. *mycoides* SC or in M. capricolum subsp*. capricolum,* respectively, while 102 have homologs in both species. Phylogenetic analysis and similarity comparisons of the 102 CDSs did not allow us to conclude whether they were more similar to M. mycoides subsp. *mycoides* SC or to M. capricolum subsp*. capricolum* (Figure S2). Additionally, one CDS (MAG4270) had a homolog only in M. mycoides subsp. *capri,* for which only a limited number of sequences are available. The occurrence of HGT was further supported by the genomic organization in M. agalactiae of 115 of the predicted transferred genes that occur as clusters containing two to 12 elements with approximately half of them displaying the same organization as in M. mycoides subsp. *mycoides* SC and M. capricolum subsp. *capricolum* genomes. Eleven of these clusters, which are distributed all over the M. agalactiae genome, are shown in Figure 2.

**Figure 3:**
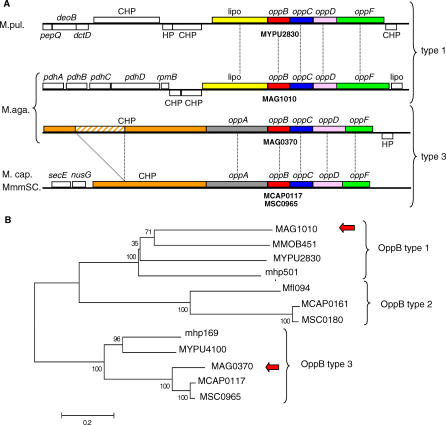
Genomic Organization and Phylogenetic Relationship of the Genes Encoding the Oligopeptide Transporter in Mycoplasmas (A) Comparison of the genomic organization of the *opp* loci in M. agalactiae, M. pulmonis, M. capricolum subsp. *capricolum,* and M. mycoides subsp. *mycoides* SC. Genes are represented by boxes positioned above or below the main line according to their relative orientation on the genome. Homologous genes are indicated by identical color; closest orthologs are connected by dashed lines. A 1,357-aa insertion in the orange-colored CHP of M. agalactiae is represented by a hatched box. CHP, conserved hypothetical protein; lipo, predicted lipoprotein; M.aga., M. agalactiae; M. cap., M. capricolum subsp. *capricolum*; MmmSC, M. mycoides subsp. *mycoides* SC; and M.pul., M. pulmonis. (B) Phylogenetic tree inferred from the amino acid sequence of OppB proteins. Bootstrap support percentages (based on 500 replicates) are indicated near each node of the tree. The two copies present in M. agalactiae are indicated by red arrows. Sequences are designated according to their mnemonic followed by their identification number as indicated in public databases. MAG, M. agalactiae; MCAP, M. capricolum subsp. *capricolum*; Mfl, *Me. florum*; mhp, M. hyopneumoniae; MMOB, M. mobile; MSC, M. mycoides subsp. *mycoides* SC; and MYPU, M. pulmonis.

As previously mentioned, 73 of the predicted transferred CDSs have an ortholog in genomes of the hominis group. In a hypothesis regarding transfer from the mycoides cluster to M. agalactiae, one might expect to detect pseudo-paralogs [37] in the M. agalactiae genome, with one inherited from an ancestor of the hominis group, while the other was acquired by HGT. Indeed, in 17 unambiguous cases, vertically and horizontally inherited pseudo-paralogs were found. As an example, the gene encoding the glucose-inhibited division protein is present as a single copy in the genomes of M. pulmonis, M. synoviae, *M. mobile,* and M. hyopneumoniae. In M. agalactiae, two copies of this gene were found; one, MAG2970, has a BBH in *M. pulmonis,* while the other, MAG1470, has a BBH in M. mycoides subsp. *mycoides* SC. The oligopeptide ABC transporter locus (*opp* genes) is another interesting example, since *opp* genes occur twice in *M. agalactiae,* at two distinct loci. As shown in Figure 3, one *opp* locus (designated as the type 1) is composed of four *opp* genes (B–D and F), the sequences of which are highly similar to those of one of the two *M. pulmonis opp* loci. The other *opp* locus of M. agalactiae (type 3, Figure 3) is composed of five *opp* genes (A–D and F), the sequences and organization of which are closer to one of the two *opp* gene loci of M. capricolum subsp. *capricolum* and M. mycoides subsp. *mycoides* SC. Phylogenetic analyses of the *oppB* genes of types 1 and 3 with homologous sequences of other mycoplasma species suggest different origins for the two *M. agalactiae opp* loci. While the type 1 was inherited from a common ancestor of the hominis branch, the type 3 was laterally acquired from the mycoides cluster. A third, isolated, copy of the *oppB* gene (MAG4700) was predicted in the M. agalactiae genome, and might represent a relic of a displaced *opp* operon, as its best orthologs were found in mycoplasmas of the hominis group.

For CDSs found only once in the genome of M. agalactiae, the situation might be more complex, as illustrated by the glycerol kinase/glycerol uptake facilitator operon, *glpK–glpF* (MAG4470–MAG4480), which was unambiguously found to originate from a mycoides ancestor (Figure S3). This operon occurs as a single copy in all mycoplasma genomes of the hominis group but is absent from M. synoviae. Because of the relative phylogenetic closeness of M. agalactiae and M. synoviae (Figure 1B), the question arises as to whether *glpK–glpF* was lost in their common ancestor and acquired later on by M. agalactiae from the mycoides cluster.

While examining M. agalactiae candidates for HGT, sequence alignments showed that 38 are truncated versions of their homologs in M. capricolum subsp. *capricolum* and M. mycoides subsp. *mycoides* SC, or were annotated as pseudogenes (Table S2 and Figure S2).

Additionally, only 14 CDSs were suspected to have undergone HGT between M. agalactiae and species of the pneumoniae phylogenetic group or non-mollicute bacteria (Table S3).

### Putative Barrier to Gene Transfer: Hyper-Variable Restriction-Modification Systems

Since restriction–modification (RM) systems serve in bacteria as a tool against invading DNA [38], it was of interest to specifically search for these systems in light of the high level of HGT in M. agalactiae. One locus encoding a putative RM system is composed of six genes with homology to type I RM systems (Figure S4) and was designated *hsd*. It contains (i) two *hsdM* genes (MAG5650 and MAG5730), coding for two almost identical modification (methylase) proteins (94% identity), which would methylate specific adenine residues; (ii) three *hsdS* genes (MAG5640, MAG5680, and MAG5720), each coding for a distinct RM specificity subunit (HsdS) that shares homology with the others (between 50% to 97% similarities); and (iii) one *hsdR* pseudo-gene (MAG5700/MAG5710), which is interrupted in the middle by a stop codon and would otherwise encode a site-specific endonuclease (HsdR). Finally, the *hsd* locus contains two hypothetical CDSs (MAG5660 and MAG5670) and one gene (MAG5690), whose product displays 76.9 % similarity to a phage family integrase of Bifidobacterium longum [39] and motifs found in molecules involved in DNA recombination and integration. In M. pulmonis, the *hsd* locus has been shown to undergo frequent DNA rearrangements but the gene encoding the putatively involved recombinase is located elsewhere on the genome [26,40].

Apart from this locus, only three other unrelated M. agalactiae CDSs display similarities with the restriction–modification system, one of which was annotated as a pseudogene.

### M. agalactiae Lipoproteins: An Extended Repertoire Including Several Lipoprotein Genes Involved in HGT with the Mycoides Cluster

Mycoplasma lipoproteins are of particular interest because they have been proposed to play a role in the colonization of specific niches and in interaction with the host [11,41]. In order to identify the putative lipoproteins encoded by the M. agalactiae genome, we combined results obtained by PS-SCAN analysis with the detection of a signature that was defined by using MEME/MAST software and a set of previously identified mycoplasma lipoproteins (see Material and Methods). This strategy resulted in the prediction of 66 lipoproteins, 85% of which were annotated as hypothetical proteins. The remaining 15% correspond to the previously characterized Vpmas, P40, P30, and P48; and to two CDSs homologous to the substrate-binding protein of an oligopeptide (OppA, MAG0380) and to an Alkylphosphonate ABC (MAG5030) transporter, respectively.

Among the genes encoding the 66 predicted lipoproteins, our analyses indicated that the corresponding genes of 19 have undergone HGT with the mycoides cluster (see Tables S2, S5, and S6). These 19 CDSs were annotated as hypothetical proteins, however, four (MAG2430, MAG3260, MAG6480, and MAG7270) share a high level of similarity, and constitute, with nine other polypeptides (MAG0210, MAG0230, MAG1330, MAG1340, MAG3270, MAG4220, MAG4310, MAG6460, and MAG6490), a protein family. A MEME/MAST analysis indicated that the 13 proteins of this family shared one to ten repeats of a 25 amino-acid motif A ([KN]W[DN][TV]SNVT[ND]MSSMFxGAK[KS]FNQ[DN][IL]S) (Figure S5). This motif is highly similar to the DUF285 domain of unknown function predicted in a large number of mycoplasma lipoproteins and found only in the mycoides cluster and in some non-mollicute bacteria (i.e., *Listeria monocytogenes, Enterococcus faecalis, Lactobacillus plantarum,* and Helicobacter hepaticus). A second motif B ([FM]PKN[VT][KV]KVPKELP[EL][EK][IV]TSLEKAFK[GN]) was also found in most of the family proteins. Of the 13 members of the family, whose corresponding genes are distributed all over the chromosome, five were predicted to be lipoproteins; the others may constitute a reservoir of sequence to generate surface variability. Altogether, these data suggest that M. agalactiae has inherited a family of genes encoding potentially variable lipoproteins that are otherwise specific to the mycoides cluster.

Another remarkable lipoprotein family is found in the portion of the genome (MAG7050–MAG7100; Figure S4) that encodes the phase-variable, related Vpma products. The Vpma family has been extensively described [29,30] and was previously shown to present typical elements of mobile pathogenicity islands [29]. However, comparison of the Vpmas coding sequences with other mycoplasma genomes indicate that they are specific of the M. agalactiae species, although their variation in expression and genetic organization closely resembles the Vsp system found in the close relative M. bovis [42–44]. No similar system or coding sequences was found in the mycoides cluster.

### ICE as Vehicles for HGT in Mycoplasmas?

To our knowledge, attempts to naturally transform M. agalactiae or other mycoplasma species have failed, suggesting that HGT, if it occurs, is mediated via another mechanism. Only a limited number of viruses or natural plasmids have been described so far in mycoplasmas that could account as vehicles for HGT, apart from a new ICE that has been described in a few Mycoplasma spp. [12,15]. In a recent study, we documented the occurrence of such an element in M. agalactiae strain 5632 (ICEA_5632_) as chromosomal multiple copies and as a free circular form [16].

**Figure 4:**
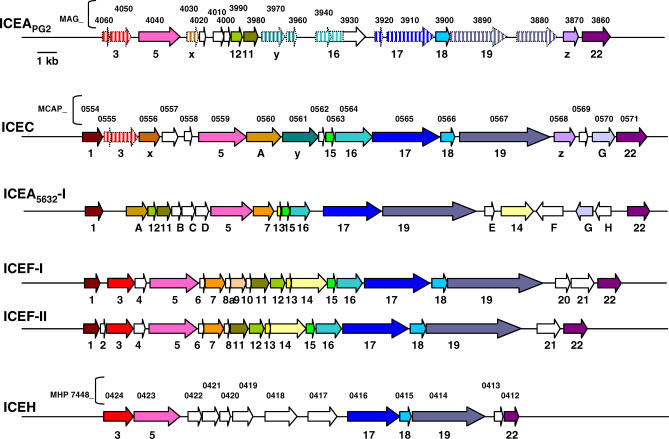
Genomic Organization of ICEs and ICE-Related Genes in Mollicutes Arrows represent CDSs, hatched arrows represent pseudogenes. Homologous CDSs are identified by the same colour and same number or letter underneath. Numbering is using the nomenclature of M. fermentans ICEs (ICEFI and II) as a reference [15], whereas letters refer to the CDSs without any ortholog in ICEF. Locus tags for genes from the ICEs of M. agalactiae strain PG2, M. capricolum subsp. *capricolum* strain California Kid, and M. hyopneumoniae strain 7448 (MAG_4060–3860, MCAP_0554–0571, and MHP7448_424– 412, respectively) are indicated above the arrows. Other ICE-related genes present in M. hyopneumoniae strain 232, M. pulmonis, M. mycoides subsp. *mycoides* SC, and S. citri are not shown on the figure. ICEA_PG2_ *,* ICE of M. agalactiae strain PG2; ICEC, ICE of M. capricolum subsp. *capricolum* strain California Kid; ICEA_5632_-I, ICE of M. agalactiae strain 5632; ICEH_7448_, ICE of *M. hyopneumoniae* strain 7448; and ICEH_232_, ICE of *M. hyopneumoniae* strain 232.

One copy, ICEA_5632_-I, was fully sequenced and Southern blot analyses suggested that it occurs in a minority of strains that did not include the PG2 type strain [16, 45]. However, detailed sequence analyses performed in this study revealed that 17 CDSs of the M. agalactiae PG2 genome display different levels of similarities to CDSs present in ICEA_5632_-I and in other ICEs (Table S4) found in M. capricolum subsp. *capricolum* (ICEC), M. fermentans (ICEF-I and –II) [15], and M. hyopneumoniae strain 7448 (ICEH) [12]. These seventeen CDSs are clustered in the PG2 genome within a unique 20-kb locus, ICEA_PG2_ (Figure 4), and those with an ortholog in M. fermentans ICEF and/or M. agalactiae ICEA_5632_-I were designated as in previous reports [15,16]. Surprisingly, best alignments for ICEA products of the PG2 strain were consistently obtained with M. capricolum subsp. *capricolum* ICEC counterparts, with an average of 40% identity and 75% similarity, whereas alignments with ICEA_5632_-I or ICEF gave lower values. This close relationship between ICEA_PG2_ and ICEC was confirmed by bootstrap values of the phylogenetic trees inferred from the amino acid sequence of TraG, TraE, ORF19, and ORF22 (Figure S6). Moreover, ICEA_PG2_ and ICEC share three homologous CDSs (noted as x, y, and z in Figure 4) lacking in ICEA_5632_-I and other ICEs. All these results indicate a close relationship between ICEA_PG2_ and ICEC, and suggest that the ICEs found in strains PG2 and 5632 have a different history.

In strain PG2, the gene encoding TraE (MAG3910/MAG3920), a major actor in DNA transport across the conjugative pore, was found to be disrupted. In addition, a total of 11 out of the 20 ICEA_PG2_-CDSs might represent pseudogenes (hatched arrows in Figure 4), due to the presence of stop codons and/or frameshifts. Finally, regions directly flanking ICEA_PG2_ do not display the typical motifs found on each side of integrated ICEF and ICEA_5632_. These data strongly suggest that ICEA_PG2_ is unlikely to be functional.

In M. agalactiae strain 5632, ICEA_5632_-I excision leads to a chromosomal site that is reorganized into an “empty” locus carrying remnant motifs that cover a 476-bp sequence [16]. Interestingly, in the PG2 chromosome, a 476-bp sequence located ∼ 270 kb upstream from ICEA_PG2_ was found that is 94% identical to the sequenced “empty” ICEA_5632_-I locus, and includes the putative remnant motifs in the same order and spacing (Figure S7). Unfinished sequence data from the strain 5632 reveals that this 476-bp sequence is actually part of a larger (∼40 kb) synthenic region between PG2 and 5632.

### HGT among Phylogenetically Remote Mycoplasma Species with Sequenced Genomes

The high number of CDSs predicted to have undergone HGT between M. agalactiae and organisms of the mycoides cluster prompted us to examine possible HGT events among other mycoplasma species whose genomes have been sequenced. For each mycoplasma genome, the CDSs with a BBH in a phylogenetic group different from that of the query were then identified (see Materials and Methods). Phylogenetic analyses, when possible, were applied to detect which, among the identified CDSs, were candidates for HGT (Table 2). Overall, this analysis clearly pointed out two cases of significant HGT levels, between the mycoides cluster and M. agalactiae and between *M. gallisepticum* and M. synoviae. Detailed examination of the data revealed a clear picture for *M. synoviae,* in which all identified CDSs but one designate M. gallisepticum as the HGT partner (Tables 2, S8, and S9). This is confirmed by the reciprocal data in *M. gallisepticum,* although in several cases the phylogeny was not strong enough to support with certainty a direct association with M. synoviae. These data are consistent with a previous study in which HGT between those two species was suspected [12]. No significant HGT was detected among other mycoplasma species across phylogenetic groups apart from that described above between M. agalactiae and mycoplasmas of the mycoides cluster (see also Tables S5 and S6).

**Table 2:**
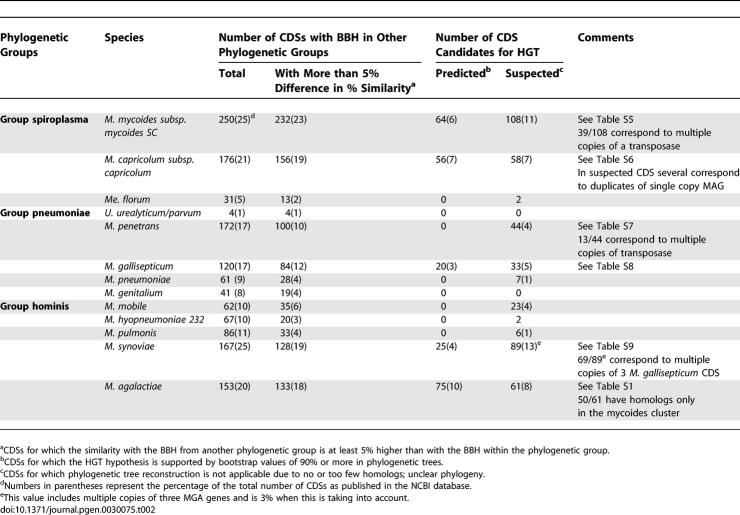
Number of Candidates for HGT among Mollicutes across the Spiroplasma, Pneumoniae, and Hominis Phylogenetic Groups

For the human mycoplasma *M. penetrans,* which has the largest genome of the dataset, a fairly large number of CDSs had BBH in a phylogenetic group other than the pneumoniae group. However, none of these candidates for HGT were confirmed by further phylogenetic analysis.

## Discussion

### Mycoplasma agalactiae Genome Has Evolved by Substantial Gene Gain

Sixteen genome sequences from different mycoplasma species are now available in public databases and provide comprehensive data for comparative genomic studies that will, for instance, contribute to the understanding of their intriguing regressive evolution (by loss of genetic material) from Gram-positive bacteria with low GC content. Indeed, mycoplasmas are thought to be fast-evolving bacteria, as supported by their positioning on some of the longest branches of the bacterial phylogenetic tree [21]. This observation is in agreement with their small genome size, and hence with their limited DNA-repair capabilities [46]. Consequently, mycoplasma genomes would be prone to accumulate mutations that would contribute to further downsizing. In this scenario, acquisition of new genes by HGT was not considered to play a major role in mycoplasma evolution. Indeed, statistical analyses predicted that the smallest proportion of HGT occurred among bacteria in symbiotic or in parasitic species, including mycoplasmas [47]. Nonetheless, a few remarkable cases of HGT involving mycoplasmas have been described that include the independent displacements of the *rpsR* and *ruvB* genes with orthologs from ɛ–Proteobacteria [48,49] and the horizontal transfer of the surface-protein VlhA encoding gene among three phylogenetically distant mycoplasmas (*M. gallisepticum, M. imitans,* and M. synoviae), which are respiratory pathogens of gallinaceous birds [50,51]. More recently, sequencing of the M. synoviae genome suggested that ∼3% of the total genome length has undergone HGT in between M. gallisepticum and M. synoviae [12]. Analyses performed in this study confirmed this trend using a different approach, which estimated that ∼3%–8 % of their CDS have been involved in HGT in between the two avian species. However, these values are much lower than the ones found for *M. agalactiae,* in which 10%–18% of its coding genome was predicted to have undergone HGT with mycoplasmas belonging to the mycoides cluster. This proportion represents, to our knowledge, the highest extent of HGT for a bacterium with a small genome size (<1 Mb). The scattering of the HGT loci all over the M. agalactiae genome suggests the occurrence of multiple HGT events and/or the shuffling via intrachromosomal recombination events of alien genes after integration. Although HGT events could be confirmed by phylogenetic analyses, it was not possible to identify significant biases in the GC composition of the transferred genes that would distinguish them from ancestral genes. It is likely that the HGT events in M. agalactiae did not take place recently and/or that the acquired sequences quickly adjusted to their new genome pattern. In fact, it has been shown that the bias in GC content is not a reliable indicator for detecting HGT events [52,53].

### HGT among Mycoplasma Species Sharing the Same Host

Demonstrating the acquisition of genes by HGT is not trivial, especially among mycoplasma species that share a number of genetic features and are phylogenetically clustered. Analyses of the M. agalactiae genome with respect to HGT with mycoplasmas of the mycoides cluster revealed roughly two categories of CDSs: one composed of CDSs with several homologs and their BBH within the mycoides cluster, and one composed of CDSs that have few or no homologs but are highly similar to CDSs of the mycoides cluster. While for the first category, phylogenetic tree reconstruction can demonstrate or refute HGT, the issue is more delicate for the second. For instance, 50 CDSs of M. agalactiae have no homolog other than in the phylogenetically distinct mycoides cluster, raising the question of whether these genes were laterally acquired from these mycoplasmas or from a third common partner that has yet to be identified. In addition, sharing the same host might have resulted in M. agalactiae and mycoplasmas of the mycoides cluster retaining a common ancestral set of genes that were lost in all other species that do not colonize ruminants. Although these alternative hypotheses cannot be formally ruled out, they all imply a series of parallel, independent events. Taking into account that *M. agalactiae,* when compared to other sequenced mycoplasmas species of the same phylogenetic group (Figure 1B), is located on one of the most ramified branches of the phylogenetic tree, this scenario seems unlikely.

The more global analyses performed on the available genomes from mollicutes (with the exception of phytoplasmas) and on M. agalactiae identified four species in which HGT has taken place. Detailed results clearly identified only two pairs of partners, each from a different phylogenetic group: (i) M. agalactiae and the mycoplasmas of the mycoides cluster, and (ii) all mycoplasma pathogens of ruminants and M. gallisepticum and M. synoviae, two pathogens of poultry. This striking observation tends to indicate that mycoplasmas sharing a common host have the capacity to exchange genetic material. These mycoplasma species are the only ones sequenced thus far that are located in different phylogenetic groups but share the same lifestyle in terms of ecological niches (Table S1).

Indeed, other sequenced species that share the same host all clustered into the same phylogenetic group (human mycoplasmas of the pneumoniae group) and therefore our approach will not detect HGT among these mycoplasmas. For one human mycoplasma, *M. penetrans,* a number of putative HGTs were found (see Tables 2 and S7) but none were supported by phylogenetic analyses. The occurrence of HGT among human mycoplasma species cannot be dismissed by this study and remains to be investigated.

### HGT and Adaptation to a Ruminant Host

A striking feature of the HGT in this bacterium is that nearly all the events were predicted to have occurred with species of the mycoides cluster, which are, with *M. agalactiae,* pathogens for ruminants. Sharing this common environment would have favored the transfer of genetic material between these mycoplasmas and the fixation of genes leading to an increased fitness as parasites of ruminants. Interestingly, ∼30 % of the genes that have undergone HGT with a mycoides ancestor correspond to membrane-associated proteins, including several transporters or lipoproteins (Table 3). As surface proteins such as lipoproteins are supposed to play a major role in the mycoplasma–host interaction, this finding supports the proposal that genes acquired by HGT may have significantly favored the colonization of ruminants by the mycoplasma. Noticeably, a family of 13 CDSs of M. agalactiae has undergone HGT with the mycoides cluster. The predicted proteins contain repeats of a domain of unknown function (DUF285). The distribution of this domain in mycoplasmal proteins is strictly restricted to species belonging to the mycoides cluster. Whether this family, which includes several lipoproteins, participates in the interaction between the mycoplasma and its ruminant host remains to be elucidated.

**Table 3:**
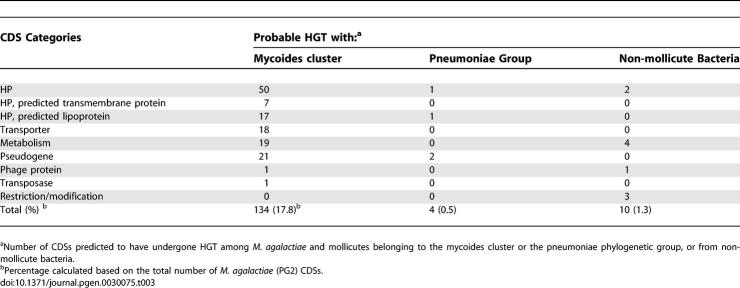
Number of M. agalactiae (PG2) CDSs Predicted to Have Undergone HGT

At present, it remains rather difficult to evaluate the selective advantage that could have provided the acquired genes, especially because half of them encode proteins with unknown functions. A possible exception could be the oligopeptide transport system Opp, of unknown specificity, for which two loci have been found in the M. agalactiae genome. In bacteria, Opp transport systems participate in a wide range of biological events including biofilm formation [54], antimicrobial-compound production [55], and adaptation to specific environments [56–58], including milk [59,60]. The substrate specificity of Opp systems is determined by the OppA subunit and it is apparent that the predicted OppA proteins from the two M. agalactiae systems do not share any sequence similarity, in contrast to the other Opp subunits. Interestingly, one of them (MAG1000) shows 44% similarity with an M. hominis ortholog that is a lipoprotein involved in adherence to host cells and proposed to be a major ATPase [61,62]. The other OppA subunit (MAG0380) shows 83% and 82% similarity with M. capricolum subsp. *capricolum* and M. mycoides subsp. *mycoides* SC OppA, respectively. Although further studies are required to determine the role of the two Opp systems in M. agalactiae, it is reasonable to propose that the Opp system inherited from the mycoides ancestor could be directly involved in the adaptation to their ruminant hosts.

In virulent M. mycoides subsp. *mycoides* SC strains, cytotoxic effects towards host cells have been correlated with the ability of the bacteria to produce high amounts of hydrogen peroxide during the catabolism of glycerol [63,64]. Glycerol is imported and phosphorylated via two alternative systems, the GlpK/GlpF system and the GtsABC transporter. The glycerol-3-phosphate enters glycolysis after oxidation by the l-alpha-glycerophosphate oxidase (GlpO); this step results in production of H_2_O_2_ as a toxic by-product.

In M. agalactiae, it is noteworthy that gene clusters encoding the GlpK/GlpF and GtsABC systems have probably been inherited from a mycoides ancestor, suggesting an ability to import glycerol for energetic metabolism. However, in *M. agalactiae,* there is no *glpO* gene upstream of *glpK/glpF,* as found in M. mycoides subsp. *mycoides* SC and M. capricolum subsp. *capricolum* genomes, or elsewhere in the genome. As in several other mollicutes, a gene encoding a glycerol-3-phosphate dehydrogenase (*gpsA*, MAG0500) is present in M. agalactiae. This suggests that M. agalactiae is able to efficiently import glycerol and to use it as a carbon and energy source but that glycerol catabolism is not coupled with H_2_O_2_ production.

### ICE and Large-Scale Gene Transfer

The mechanism of gene transfer among ruminant mycoplasmas remains to be elucidated but some recently published data raise interesting possibilities. Indeed, an ICE has been described in M. agalactiae strain 5632 [16] and a decayed ICE is also predicted in strain PG2 (ICEA_PG2_). ICEs, also designated CONSTINS or conjugative transposons, are widespread amongst prokaryotes, and are viewed as modular scaffolds with diverse genetic organizations and encoded functions that are able to confer various metabolic or resistance traits to their host and to disseminate in bacterial populations [65].

A striking feature is that ICEA_PG2_ CDS products displayed a closer degree of relatedness with the M. capricolum subsp. *capricolum* ICEC than with the M. agalactiae ICEA_5632_-I, which appeared to be more related to M. fermentans ICEF. The finding of higher sequence similarity between ICEC and ICEA_PG2_ suggested that these elements are or have been functional for lateral gene transfer between the mycoides cluster and M. agalactiae ancestors. The finding in PG2 of a sequence that is known to be generated by excision of the ICEA_5632_-I in strain 5632 can be viewed as a trace of a past excision or as a mere potential integration site for an ICE. Although mycoplasma ICEs display a modular structure and some species-to-species variations, they constitute a very homogenous set of genetic elements that appear to be specific to this class of bacteria. In particular, certain conserved CDSs (CDS19 or CDS22) that are present in all sequenced mycoplasma ICEs (Figure 4) do not have any homologs outside of the mollicutes. Only CDS5 and CDS17 have homologs (TraG and TraE, respectively) in certain other ICEs or conjugative plasmids.

Insertion sequences (ISs) are another type of mobile element that may be involved in genome plasticity. The IS IS*Mag1* was identified in several strains of M. agalactiae [66] and has most probably been exchanged between strains of M. agalactiae and M. bovis [67]. Although a complete IS*Mag1* could not be found in the sequenced genome from strain PG2, sequence analysis revealed that a fragment of this IS is located between positions 391476 and 391626. Moreover, other ISs are shared by M. bovis and M. mycoides subsp. *mycoides* SC, suggesting again that HGT events occur between these ruminant mycoplasmas [67]. Finally, our analysis of the M. agalactiae genome also revealed two genes encoding a putative prophage protein (MAG6440) and a phage family integrase (MAG5690). All of these genetic elements can be regarded as vestiges of potential shuttles that may have been involved in the transfer of genome fragments between ancestors of M. agalactiae and of mycoplasmas of the mycoides cluster.

As mentioned earlier, it is not known whether mycoplasmas are naturally competent and whether they can uptake naked DNA in their host environment. Viruses or natural plasmids that could serve as vehicles for HGT have thus far been described only in a limited number of mycoplasma species that do not include M. agalactiae. The presence of conjugative elements in M. agalactiae and in a phylogenetically distant member of the mycoides cluster, together with evidence of large-scale gene transfer in between those species strongly suggests that these simple organisms are being “sexually competent,” most likely via a conjugation-like mechanism.

Overall, data obtained in this study shed new light on the phenomenon that may underline the plasticity and evolution of mycoplasma genomes. While members of the genus *Mycoplasma* infect a wide range of hosts, individual species are thought to have strict host specificity. However, examples that have recently emerged from the literature may begin to challenge this idea [68]. Two examples are the isolation of the human-infecting mycoplasma M. fermentans from small ruminants [69] and the discovery in birds of M. capricolum-like strains closely related to the ruminant pathogen M. capricolum subsp. *capricolum* [70]. Whether HGT plays a role in adaptation to new hosts or in virulence has yet to be discovered, but understanding the mechanisms underlying HGT in mycoplasmas and their role in reshaping their reduced genomes is the next, exciting challenge.

## Materials and Methods

### Mycoplasma strain and DNA isolation.

The M. agalactiae PG2 type strain was originally isolated from a goat in Spain (1952). In previous studies, the PG2 strain was shown to contain a locus designated as *vpma* that encodes a family of abundant related lipoproteins [30] and that undergoes frequent DNA rearrangements [29]. The entire *vpma* locus was previously sequenced for the 55–5 clonal variant from the PG2 strain [29]; this variant was selected in this study for genome sequencing. The 55–5 clone was propagated in SP4 medium [71] at 37 °C. Genomic DNA was isolated as previously described [72].

### Genomic libraries, shotgun sequencing, contigs assembly, and finishing.

Three genomic libraries were constructed for sequencing purposes. Two were obtained by mechanical shearing of *M. agalactiae* total DNA and subsequent cloning of the resulting 3–4–kb and 8–10–kb inserts into plasmids pcDNA2.1 (Invitrogen, http://www.invitrogen.com) and pCNS (pSU18 derived), respectively. From these libraries, DNA inserts from approximately 5,700 and 1,500 clones, respectively, were sequenced from both ends. For the third library, DNA fragments of ∼20 kb were generated by partial Sau3A digestion and introduced into the miniBAC plasmid pBBc (pBeloBac11 derived). From this library, DNA inserts of approximately 1,100 clones were sequenced from both ends. Plasmid DNAs were purified and end-sequenced using dye-terminator chemistries on ABI3700 sequencers (Applied Biosystems, https://www2.appliedbiosystems.com). About 16,800 reads led to an average 12-fold coverage. The Phred/Phrap/Consed software package was used for sequence assembly and quality assessment [73–75]. About 380 additional reactions were necessary to complete the genomic sequence. The integrity of the assembly was confirmed by comparing the in silico restriction map with restricted DNA fragments (SmaI, XhoI, and EclXI) previously analysed by PFGE [76].

### Identification of genetic elements and annotation.

The genome annotation was performed using the CAAT-Box platform [77], which was customized to facilitate the annotation process. CDSs were first detected using the Genemark software [78], implemented in the CAAT-Box environment. Putative CDSs of more than 300 amino acids were used to train the Markov model (order 5). The three codons AUG, UUG, and GUG were used as potential start codons, whereas UAG and UAA were defined as stop codons. Once trained, the Markov model was applied to the complete genome using 80 bp as a cut-off value for the smallest CDSs. Prediction of CDSs with CAAT-Box also integrates results of BLAST searches [79] in order to discriminate highly probable CDSs from false ORFs. Databases used for this purpose were SwissProt (http://www.ebi.ac.uk/swissprot/index.html), trembl (http://www.ebi.ac.uk/embl/index.html), and MolliGen (http://cbi.labri.fr/outils/molligen), a database dedicated to the comparative genomics of mollicutes. In order to determine the extent of sequence similarity, alignments between predicted proteins and best BLAST-hit sequences were performed using the NEEDLE software [80] implementing the Needleman-Wunsch global alignment algorithm and using the BLOSUM62 matrix. During the annotation process, proteins were considered to be homologs when the similarity in these alignments exceeded 40%. Predicted proteins with lower or only local similarities with previously characterized proteins were annotated as hypothetical proteins. Start codons were most often chosen according to CAAT-Box recommendations that resulted from both Genemark coding state prediction and BLAST results analysis. For CDSs showing neither obvious homology relationships nor clear coding curves, the most upstream start was chosen, with a preference for the most frequently used AUG codon.

Other tools incorporated into CAAT-Box were also used to improve annotation and function predictions: among them, InterProScan [81] and PrositeScan [82] for domains detection and TMHMM for trans-membrane segments prediction [83]. In order to recover small CDS or gene fragments that could have been discarded during the CDS prediction process, intergenic sequences of more than 80 bp were systematically compared to reference databases using BLASTX. The annotation of each CDS was manually verified by at least two annotators.

The tRNAs were located on the chromosome using the tRNAscan software [84] and the rRNA genes were searched using BLASTN by homology with the rRNA genes from M. pulmonis [26]. Precise boundaries were established after comparisons with the sequences stored in the European Ribosomal RNA Database (http://www.psb.ugent.be/rRNA) [85] and the 5S Ribosomal RNA Database (http://www.man.poznan.pl/5SData) [86].

### Mollicute phylogenetic analyses based on 16S rDNA sequences or on selected shared proteins for species with sequenced genomes.

For phylogenetic analyses based on 16S rDNA sequences, aligned 16S rDNA sequences were recovered from the RDPII database (release 9.46; [87]). From this alignment, 649 sites were informative. Phylogenetic analyses were performed using MEGA3 [88]. The three methods implemented in the version 3.1 of this integrated software were used: Neighbor-joining, Minimal Evolution, and Maximum Parsimony. The reliability of the tree nodes was tested by performing 500 bootstrap replicates.

For phylogenetic analyses based on selected shared proteins for species with sequenced genomes, supertree constructions were obtained using 30 shared proteins (COGs; [89]). These were selected because they have been shown not to be horizontally transferred in a large dataset [21]. Protein sequences corresponding to the selected COG (Table S10) were retrieved from the 17 mollicute genomes available in the MolliGen database. These are *M. agalactiae; Mycoplasma capricolum* subsp. *capricolum*; Mycoplasma mycoides subsp. *mycoides* SC; *Mesoplasma florum; Ureaplasma urealyticum/parvum*; *Mycoplasma penetrans; Mycoplasma gallisepticum*; *Mycoplasma pneumoniae; Mycoplasma genitalium; Mycoplasma mobile*; Mycoplasma hyopneumoniae strains 232, 7448, and J; *Mycoplasma pulmonis; Mycoplasma synoviae;* Onion yellows phytoplasma; and Aster yellows witches-broom phytoplasma. Separate multiple sequence alignments of each COG were built for all 17 mollicute genomes using ClustalW. These individual alignments were manually concatenated, which resulted in a super matrix with 5,257 informative sites. Phylogenetic analyses were performed using MEGA3 [88]. The three methods implemented in the version 3.1 of this integrated software were used: Neighbor-joining, Minimal Evolution, and Maximum Parsimony. The substitution matrix used was JTT, the sites with gaps were ignored, and the reliability of the tree nodes was tested by performing 500 bootstraps replicates. We also derived Maximum Likelihood phylogenetic inferences using PhyML [90], applying the JTT matrix and other options as reported by others [21]. The sites with gaps were ignored and support for the hypothesis of relationships was assessed using 100 bootstrap replicates.

### Lipoprotein prediction.

To identify M. agalactiae putative lipoproteins, two methods were used. In the first one, M. agalactiae CDSs were scanned for the presence of the PROSITE Prokaryotic membrane lipoprotein lipid attachment site motif (PROKAR_LIPOPROTEIN), the sequence of which is [DERK](6)-[LIVMFWSTAG](2)-[LIVMFYSTAGCQ]-[AGS]-C. Twenty-five CDSs were determined to encode lipoproteins using this method. Because some already characterized lipoproteins of M. agalactiae did not match this motif [30], a second approach was devised using MEME/MAST [91,92]. Specific motifs within the first 35 amino acids of a set of 14 characterized lipoproteins from M. agalactiae (six sequences) and other mycoplasmas (eight sequences) were analyzed using MEME. This first step resulted in the identification of two motifs, corresponding to the charged N terminus and to the lipobox. These motifs were then searched for in all of the M. agalactiae CDSs using MAST, resulting in a set of 42 proteins displaying one or both of the two motifs. Six proteins were excluded from this set because the motifs were located too far from the N terminus of the polypeptides. A second round of MEME/MAST motif search was performed using the N-terminal sequence of the 36 remaining proteins as a seed. A total of 81 CDSs were recovered. Of the recovered CDSs, those without (i) motifs located in the N-terminal region, (ii) cysteine within the lipobox motif, or (iii) charged amino acids in the N-terminus were eliminated. After a manual check, a total of 55 proteins were predicted to be lipoproteins using this method. These proteins display a region that is composed of an N-terminal sequence of 3–10 positively charged amino acids followed by a hydrophobic segment of 10–17 aa, from which K, D, R, E, and H are excluded. At the very end there is a lipobox of 4 aa, the consensus sequence of which is either (V/I)AAKC (Type KC) or (I/L)(A/S)ASC (Type SC). Finally, combining PS-SCAN and MEME/MAST methods, a total of 66 lipoproteins were predicted, among which 14 were detected by both methods. Interestingly, none of the CDS exhibiting a “KC” lipobox was detected by PS-SCAN.

### Detection of HGT in mollicute genomes.

Best Blast Hits (BBH) were identified for every predicted protein using a BLASTP threshold E-value of 10^−8^. Five databases were searched: UniProt [93], and four other databases consisting of proteomes predicted from sequenced genomes of mollicute species belonging to distinct phylogenetic groups; spiroplasma (M. capricolum subsp. *capricolum*, M. mycoides subsp. *mycoides* SC, *Me. florum* and *S. citri*), Pneumoniae (*U. urealyticum/parvum*, *M. penetrans, M. gallisepticum*, M. pneumoniae and *M. genitalium*), hominis (M. mobile, M. hyopneumoniae strain 232, M. pulmonis and *M. synoviae*), and phytoplasmas (Onion yellows phytoplasma and Aster yellows witches-broom phytoplasma).

E-values were automatically compared and data were filtered to identify putative horizontal transfers. CDSs displaying a BBH with a mollicute sequenced genome belonging to a phylogenetic group other than that of the query were further analysed as follows. Pairwise alignments [94] between each query protein and the best hits from UniProt and each of the four mollicute databases were calculated. From these alignments, the percentage of similarity of the query with its BBH obtained with mollicutes belonging to the same phylogenetic group was compared to that obtained with mollicutes belonging to a different phylogenetic group. A 5% difference in favour of an ortholog not belonging to the query phylogenetic group was considered as the minimal threshold for further investigations.

Protein phylogeny tree reconstructions were performed using the MEGA3 software [88]. Trees were obtained using the distance/neighbor-joining method and the gaps complete deletion option; bootstrap statistical analyses were performed with 500 replicates. Bootstrap values lower than 90% were not considered to be significant. When supported by significant bootstrap values, incongruence between protein and species phylogenies was understood as a potential HGT.

When very few homologs were identified or when branches were only supported by low bootstrap values, the possibility of an HGT was not recorded except when other independent results support it. These were a particularly high similarity value (>80%) and conservation of gene synteny.

## Supporting Information

Figure S1 Similarity of the CDSs from M. agalactiae and M. hyopneumoniae with Their BBH in M. pulmonis and M. capricolum subsp. *capricolum*
(54 KB PDF)

Figure S2 Similarity of the 136 M. agalactiae CDSs with their BBH in M. mycoides subsp*. mycoides* SC and M. capricolum subsp. *capricolum*
(18 KB PDF)

Figure S3 Phylogenetic Tree Inferred from the Amino Acid Sequence of GlpK Proteins (4 KB PDF)

Figure S4 Schematic Representing the Genetic Organization of Two Remarkable Loci of M. agalactiae
(13 KB PDF)

Figure S5 Schematic Representation of the 13 M. agalactiae Proteins Containing the DUF285 Domain (3 KB PDF)

Figure S6 Phylogenetic Tree Inferred from the Amino Acid Sequence of TraG, TraE, ORF19, and ORF 22 Proteins (27 KB PDF)

Figure S7 Alignment of the ICEA_5632_ Locus from M. agalactiae Strain 5632 with a Synthenic Region from M. agalactiae PG2 (6 KB PDF)

Table S1 Common Hosts and Tissue Tropisms for *Mycoplasma, Ureaplasma* Species, and Phytoplasmas with sequenced genomes (35 KB DOC)

Table S2 CDS Candidates for HGT among M. agalactiae and mycoplasmas of the mycoides cluster, M. mycoides subsp. *mycoides* and *M. capricolum* subsp. *capricolum*
MAG, *M. agalactiae;* MCAP, M. capricolum subsp. *capricolum;* MSC, M. mycoides subsp. *mycoides* SC.
(259 KB DOC)

Table S3 CDS Candidates for HGT among M. agalactiae and Mycoplasmas of the Pneumoniae Group or Non-mollicute Bacteria MAG, *M. agalactiae.*
(39 KB DOC)

Table S4 Fastap Alignments of ICEA_PG2_ CDS Products with their Homologs in ICEC, ICEA_5632_-I, and ICEF (84 KB DOC)

Table S5 CDS Candidates for HGT between M. mycoides subsp. *mycoides SC* and M. agalactiae
MAG, *M. agalactiae;* MSC, M. mycoides subsp. *mycoides* SC.
(250 KB DOC)

Table S6 CDS Candidates for HGT between M. capricolum subsp. *capricolum* and M. agalactiae
MAG, *M. agalactiae;* MCAP, M. capricolum subsp. *capricolum.*
(191 KB DOC)

Table S7 CDS Candidates for HGT between M. penetrans and Other Mollicutes MYPE, *M. penetrans.*
(80 KB DOC)

Table S8 CDS Candidates for HGT between M. gallisepticum and *M. synoviae* or Other Mollicutes MGA, *M. gallisepticum;* MS, M. synoviae.
(102 KB DOC)

Table S9 CDS Candidates for HGT between M. synoviae and *M. galliepticum*
MGA, *M. gallisepticum;* MS, M. synoviae.
(149 KB DOC)

Table S10 List of the 30 Shared Proteins Used for Supertree Construction of Figure 1B (36 KB DOC)

### Accession Numbers

The genome sequence from M. agalactiae PG2 strain, as well as related features, were submitted to the EMBL (http://www.ebi.ac.uk/embl), GenBank (http://www.ncbi.nih.gov/Genbank/index.html), and DDBJ databases (http://www.ddbj.nig.ac.jp) under accession number CU179680. All data are also available from the MolliGen database (http://cbi.labri.fr/outils/molligen).

The National Center for Biotechnology Information (NCBI) (http://www.ncbi.nlm.nih.gov) accession numbers of other genomes mentioned in this manuscript are: M. capricolum subsp. *capricolum*, NC_007633; M. gallisepticum, NC_004829; M. genitalium, NC_000908; M. hyopneumoniae 232, NC_006360; M. hyopneumoniae 7448, NC_007332; M. hyopneumoniae J, NC_007295; M. mobile, NC_006908; M. mycoides subsp. *mycoides* SC, NC_005364; M. penetrans, NC_004432; M. pneumoniae, NC _000912; M. pulmonis, NC_002771; M. synoviae, NC_007294; *Me. florum*, NC_006055; and *U. urealyticum/parvum*, NC_002162.

The NCBI locus tags of the genes and gene products mentioned in this manuscript are M. capricolum subsp. *capricolum* OppA, MCAP_0116; *M. hyopneumoniae gidA*, mhp003; *M. mobile gidA,* MMOB1540; M. mycoides subsp. *mycoides* SC OppA, MSC_0964; M. mycoides subsp. *mycoides* SC GlpF, MSC_0257; *M. mycoides* subsp. *mycoides* SC GlpK, MSC_0258; M. mycoides subsp. *mycoides* SC GlpO, MSC_0259; M. mycoides subsp. *mycoides* SC GtsABC transporter components, MSC_0516/MSC_0517/ MSC_0518; *M. pulmonis gidA,* MYPU_2530; and *M. synoviae gidA,* MS53_0515.

The Pfam database (http://www.sanger.ac.uk/Software/Pfam) accession numbers for the protein motifs/domains mentioned in this paper are phage integrase motif, PF00589; and DUF285 domain of unknown function, PF03382.

The PROSITE database (http://www.expasy.ch/prosite) accession number for the prokaryotic membrane lipoprotein lipid attachment site motif is PS51257.

## Abbreviations

BBH: best BLAST hit
HGT: horizontal gene transfer
ICE: integrative conjugative element
IS: insertion sequence
RM: restriction–modification
