# Supplementary material for: Being Pathogenic, Plastic, and Sexual while Living with a Nearly Minimal Bacterial Genome
Source: PLoS Genet. 2007 May 18;3(5):e75. doi: 10.1371/journal.pgen.0030075 (PMC1868952; doi:10.1371/journal.pgen.0030075)
Supplement: Supplementary file 1 [file pgen.0030075.sg001.pdf]

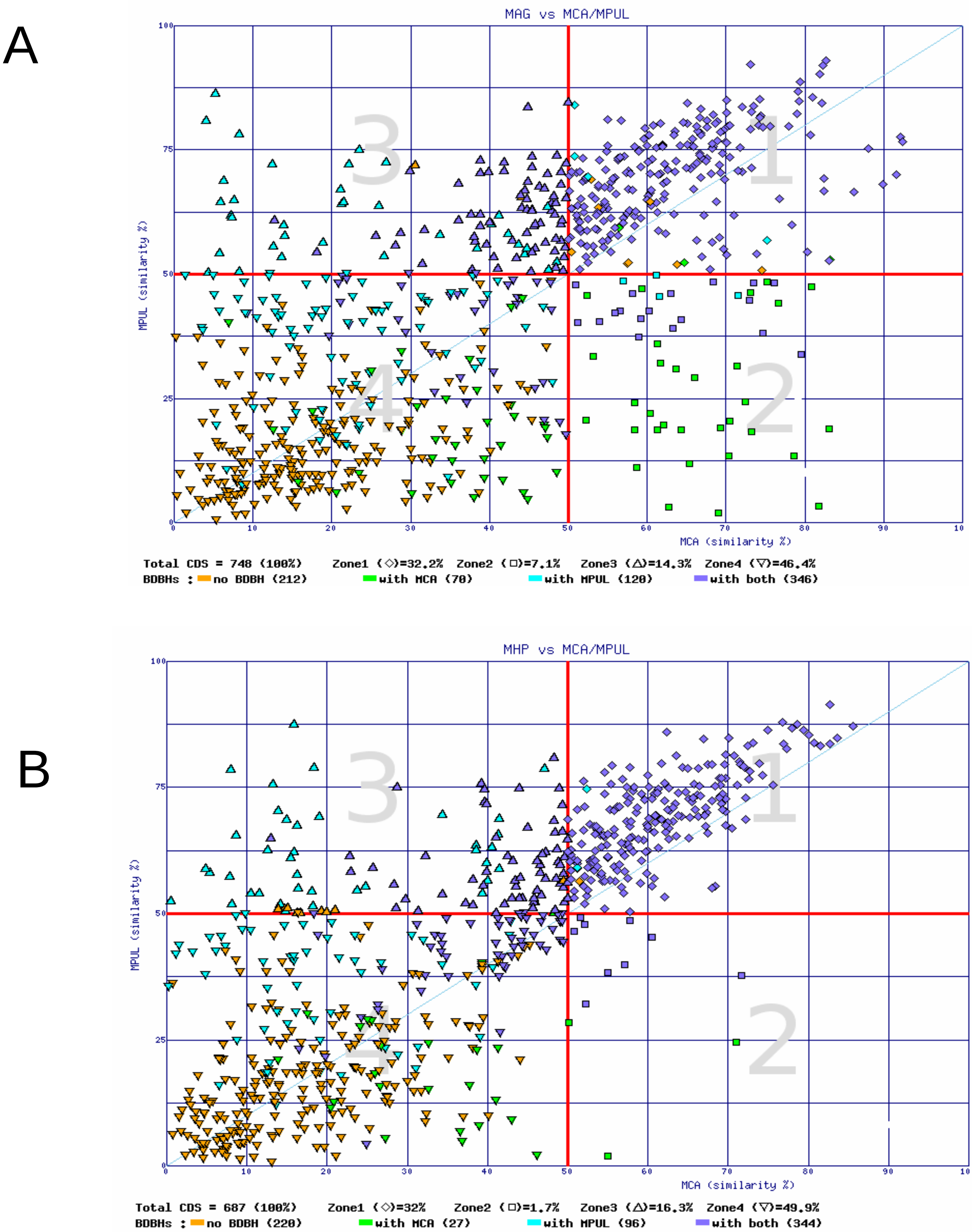

Figure S1. Similarity of the CDSs from *M. agalactiae* (A) and *M. hyopneumoniae* (strain 232) (B) with their BLAST best hits in *M. pulmonis* and *M. capricolum* subsp. *capricolum*. Each CDS from *M. agalactiae/M. hyopneumoniae* is represented as a dot positioned in function of the percentage of similarity (amino acid sequence, global alignment) with its BLAST best hit in *M. pulmonis* and *M. capricolum* subsp. *capricolum*. (A) In zone 1, most dots are positioned above the diagonal line, which indicates a closer proximity of the corresponding *M. agalactiae* CDSs with their *M. pulmonis* Bi Directional Best Hit (BDBH). However, many other CDSs appear much similar to their *M. capricolum* subsp. *capricolum* BDBH (zone 1, violet symbols under the diagonal line); some others only have a BDBH in *M. capricolum* subsp. *capricolum* (zone 2, green symbols). (B) In contrast with what was observed for *M. agalactiae*, nearly all dots are positioned above the diagonal line, which indicates a closer proximity of the corresponding CDSs of *M. hyopneumoniae* with their *M. pulmonis* BDBH. MPUL, *M. pulmonis*; MCA, *M. capricolum* subsp. *capricolum*. Detail of the symbols are indicated under the graph.
