# Supplementary material for: Being Pathogenic, Plastic, and Sexual while Living with a Nearly Minimal Bacterial Genome
Source: PLoS Genet. 2007 May 18;3(5):e75. doi: 10.1371/journal.pgen.0030075 (PMC1868952; doi:10.1371/journal.pgen.0030075)
Supplement: Supplementary file 2 [file pgen.0030075.sg002.pdf]

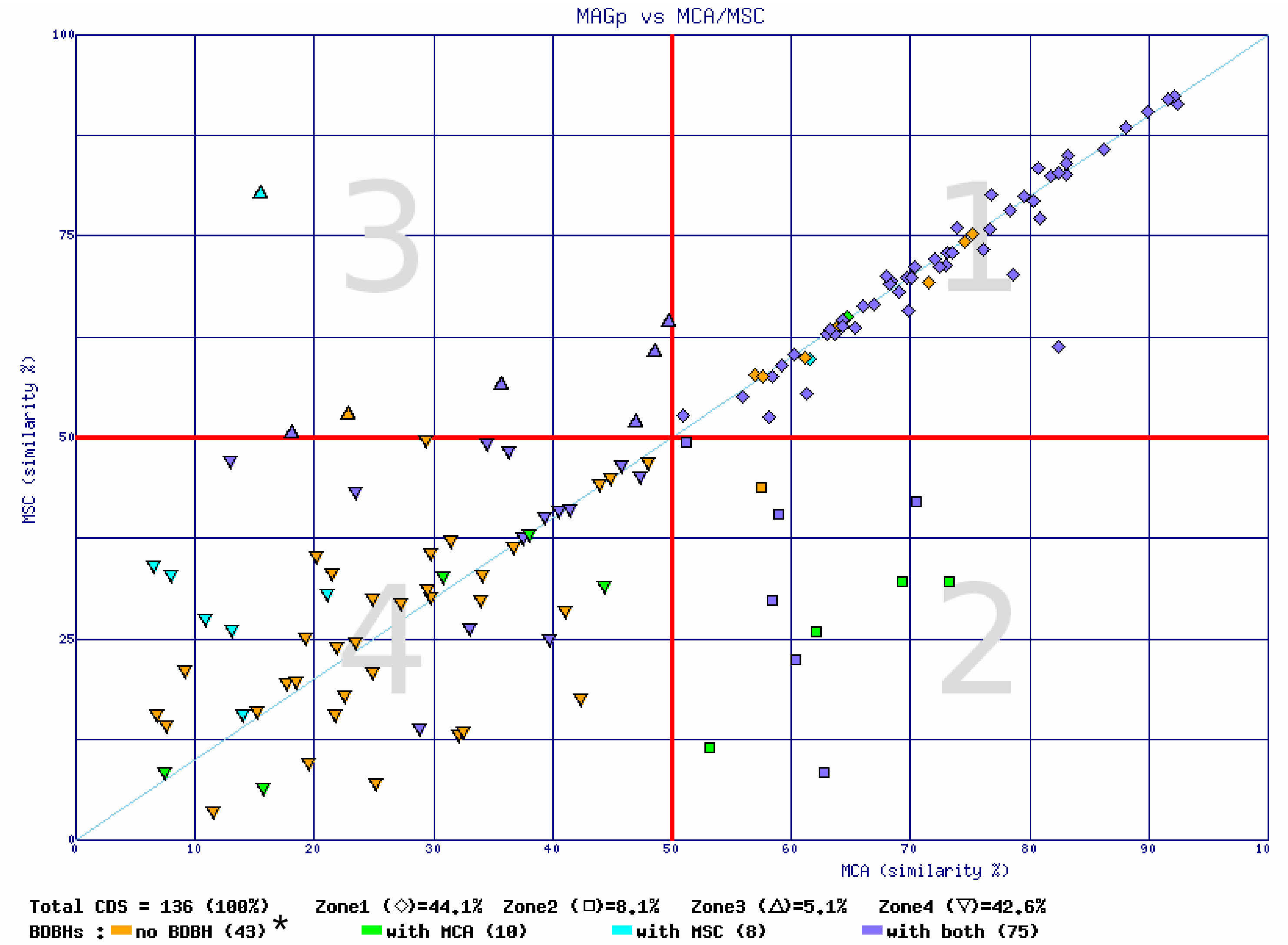

Figure S2. Similarity of 136 *M. agalactiae* CDSs with their best BLAST hits in *M. mycoides* subsp. *mycoides* SC and *M. capricolum* subsp. *capricolum*. Each of the 136 CDS from *M. agalactiae* inherited from the mycoides cluster is represented as a dot positioned in function of the percentage of similarity (amino acid sequence, global alignment) with its best BLAST hit in *M. mycoides* subsp. *mycoides SC* and *M. capricolum* subsp. *capricolum*. Most dots are positioned on the diagonal line, which indicates a similar proximity of the corresponding CDSs with its Bi Directional Best Hit (BDBH) in *M. mycoides* subsp. *mycoides* SC and *M. capricolum* subsp. *capricolum* BDBH. However, some CDSs have only a BDBH in *M. mycoides* subsp. *mycoides* SC (blue symbol) or in *M. capricolum* subsp. *capricolum* (green symbol)., MSC, *M. mycoides* subsp. *mycoides* SC; MCA, *M. capricolum* subsp. *capricolum*. Detail of the symbols are indicated under the graph.

* 38 correspond to truncated products
