# Supplementary material for: Being Pathogenic, Plastic, and Sexual while Living with a Nearly Minimal Bacterial Genome
Source: PLoS Genet. 2007 May 18;3(5):e75. doi: 10.1371/journal.pgen.0030075 (PMC1868952; doi:10.1371/journal.pgen.0030075)
Supplement: Supplementary file 3 [file pgen.0030075.sg003.pdf]

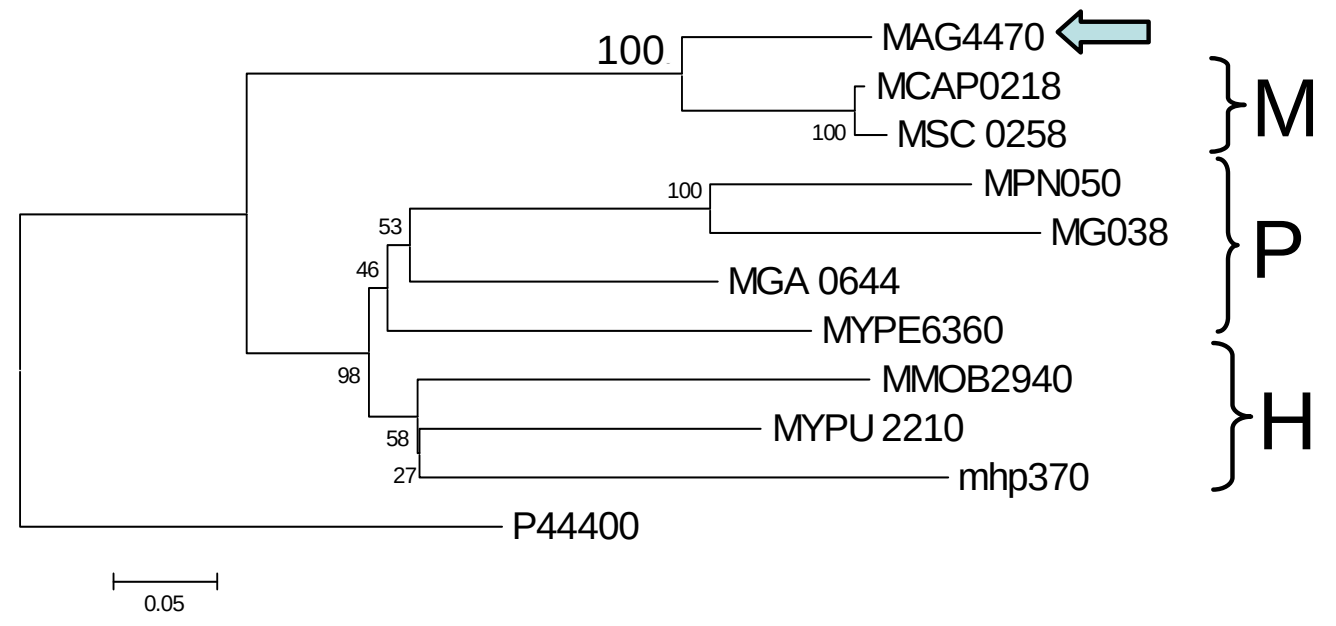

Figure S3. Phylogenetic tree inferred from the amino acid sequence of GlpK proteins. Bootstrap support values (based on 500 replicates) are indicated near each node of the tree. The GlpK protein from *M. agalactiae* is indicated by an arrow. Sequences are designated according to their mnemonic followed by their identification number as indicated in public databases. MAG, *M. agalactiae*; MCAP, *M. capricolum* subsp. *capricolum*; MSC, *M. mycoides* subsp. *mycoides* SC; MPN, *M. pneumoniae*; MG, *M. genitalium*; MGA, *M. gallisepticum*; MYPE, *M. penetrans*; MMOB, *M. mobile*; MYPU, *M. pulmonis*; mhp, *M. hyopneumoniae.* The GlpK protein from *Haemophilus influenzae* (P44400) was chosen as an outgroup.
