# Supplementary material for: Being Pathogenic, Plastic, and Sexual while Living with a Nearly Minimal Bacterial Genome
Source: PLoS Genet. 2007 May 18;3(5):e75. doi: 10.1371/journal.pgen.0030075 (PMC1868952; doi:10.1371/journal.pgen.0030075)
Supplement: Supplementary file 4 [file pgen.0030075.sg004.pdf]

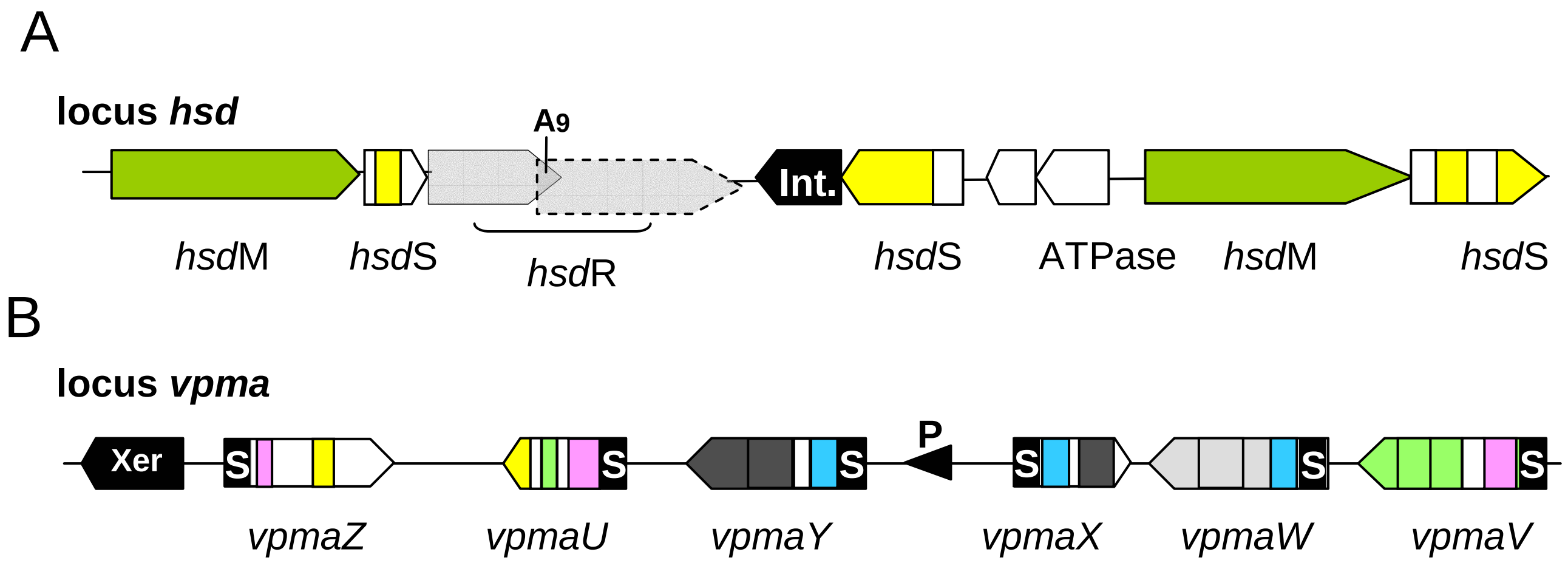

Figure S4. Schematic representing the genetic organization of two remarkable loci of *M. agalactiae*.
A. Genomic organization of the *hsd* (Host Specificity of DNA) locus that contains genes encoding a Type I restriction-modification system. The three subunits of the system are encoded by the genes *hsd*M, *hsd*S et *hsd*R. The reading frame of the gene *hsdR* is interrupted by a frameshift mutation located within a polyA (A9) track. The locus also contains a CDS (int) displaying similarity with a phage family integrase of *Bifidobacterium longum* [1] and motifs found in molecules involved in DNA recombination and integration. Identical colors (apart from white) indicate amino acid sequences with high level of similarity (between 50 to 97%).
B. Genomic organization of the *vpma* (Variable Protein of *M. agalactiae*) locus that contains a family of 6 genes encoding antigenic surface lipoproteins with sequence similarities. This locus has been extensively described [2, 3]. It undergoes frequent genomic rearrangements performed by the recombinase Xer and contains a unique promoter (P) that drives the expression of *vpmaY* in the clone sequenced in this study. Identical colors (apart from white) indicate amino acid sequences that shares homologies with each other. Sequences encoding a signal peptide are represented in black and are indicated by “S”.

1. Schell MA, Karmirantzou M, Snel B, Vilanova D, Berger B, et al. (2002) The genome sequence of *Bifidobacterium longum* reflects its adaptation to the human gastrointestinal tract. Proc Natl Acad Sci U S A 99: 14422-14427.
2. Glew MD, Papazisi L, Poumarat F, Bergonier D, Rosengarten R, et al. (2000) Characterization of a multigene family undergoing high-frequency DNA rearrangements and coding for abundant variable surface proteins in *Mycoplasma agalactiae*. Infect Immun 68: 4539-4548.
3. Glew MD, Marenda M, Rosengarten R, Citti C (2002) Surface diversity in *Mycoplasma agalactiae* is driven by site-specific DNA inversions within the vpma multigene locus. J Bacteriol 184: 5987-5998.
