# Supplementary material for: Being Pathogenic, Plastic, and Sexual while Living with a Nearly Minimal Bacterial Genome
Source: PLoS Genet. 2007 May 18;3(5):e75. doi: 10.1371/journal.pgen.0030075 (PMC1868952; doi:10.1371/journal.pgen.0030075)
Supplement: Supplementary file 5 [file pgen.0030075.sg005.pdf]

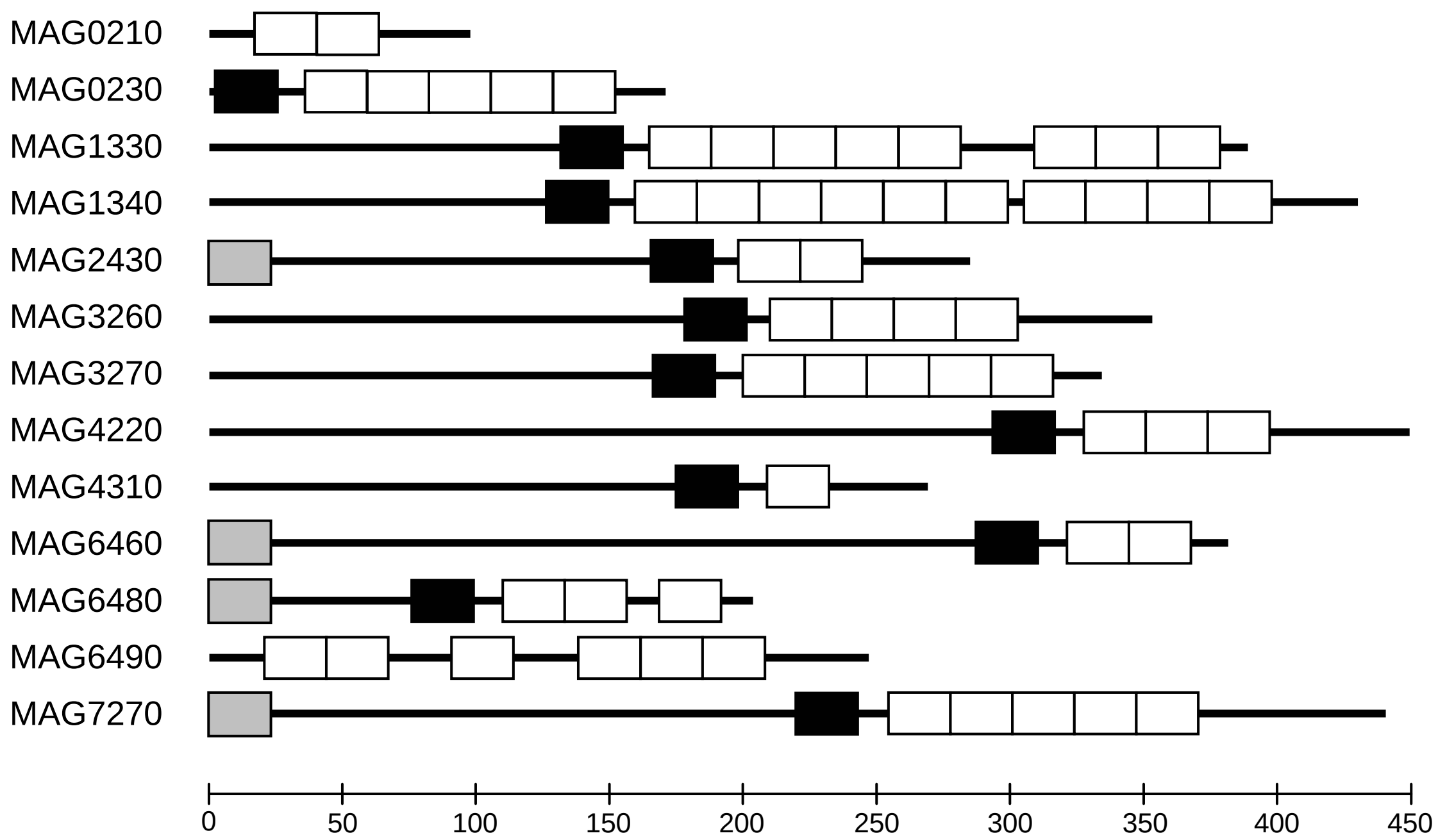

Figure S5. Schematic representation of the 13 *M. agalactiae* proteins containing the DUF285 domain. Motif A, which correspond to DUF285, and motif B are represented by white and black boxes, respectively. Four members of the DUF285 protein family were predicted as lipoproteins; the signal peptide is symbolized by a grey box. Protein length scale (in amino acid) is indicated.
