# Supplementary material for: Being Pathogenic, Plastic, and Sexual while Living with a Nearly Minimal Bacterial Genome
Source: PLoS Genet. 2007 May 18;3(5):e75. doi: 10.1371/journal.pgen.0030075 (PMC1868952; doi:10.1371/journal.pgen.0030075)
Supplement: Supplementary file 6 [file pgen.0030075.sg006.pdf]

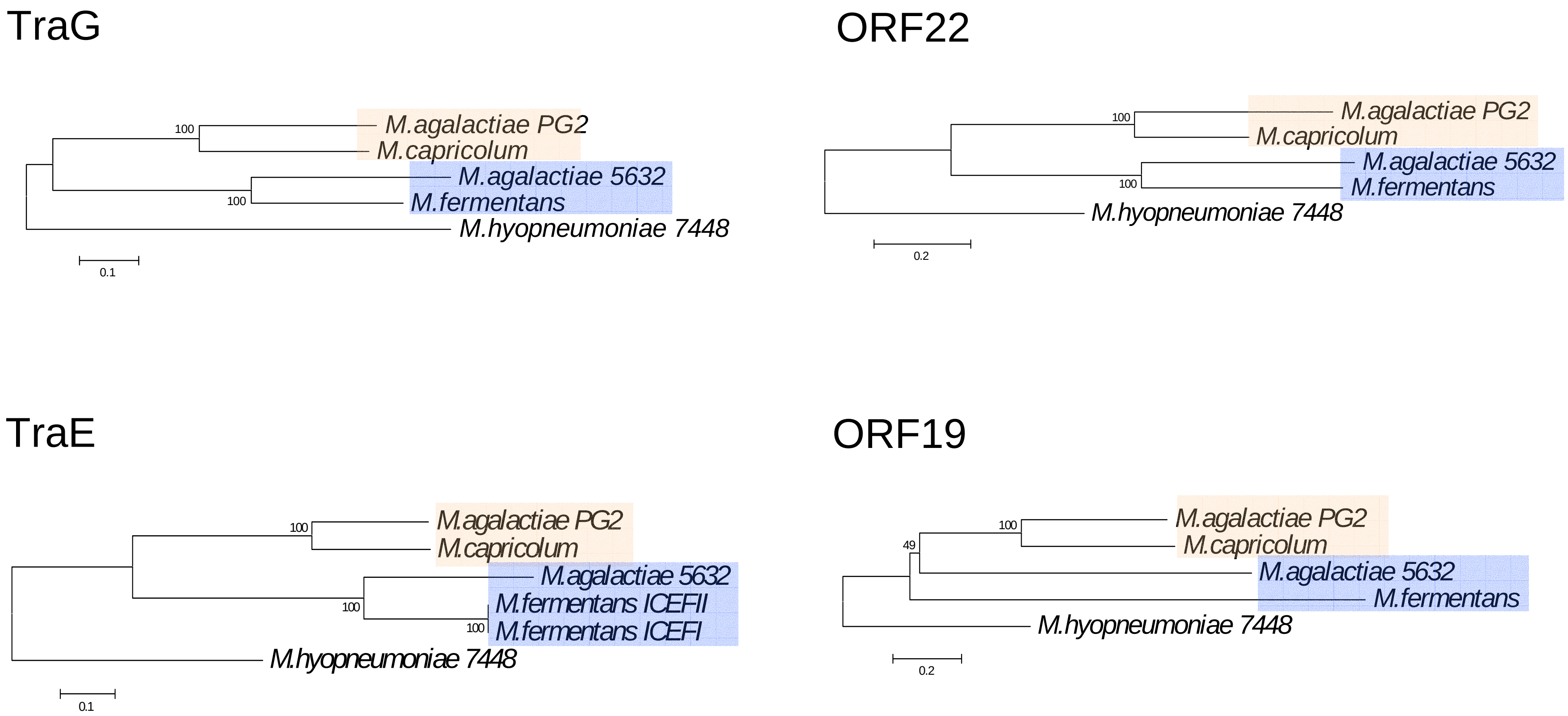

Figure S6. Phylogenetic tree inferred from the amino acid sequence of TraG, TraE, ORF19 and ORF 22 proteins. Bootstrap support values (based on 500 replicates) are indicated near each node of the tree. The proteins from *M. hyopneumoniae 7448* were chosen as outgroups. The two CDSs corresponding to the degraded genes *traE* (MAG3910/MAG3920) and ORF19 (MAG3880/MAG3890) were considered to generate the multiple alignments.
