# Supplementary material for: Being Pathogenic, Plastic, and Sexual while Living with a Nearly Minimal Bacterial Genome
Source: PLoS Genet. 2007 May 18;3(5):e75. doi: 10.1371/journal.pgen.0030075 (PMC1868952; doi:10.1371/journal.pgen.0030075)
Supplement: Supplementary file 7 [file pgen.0030075.sg007.pdf]

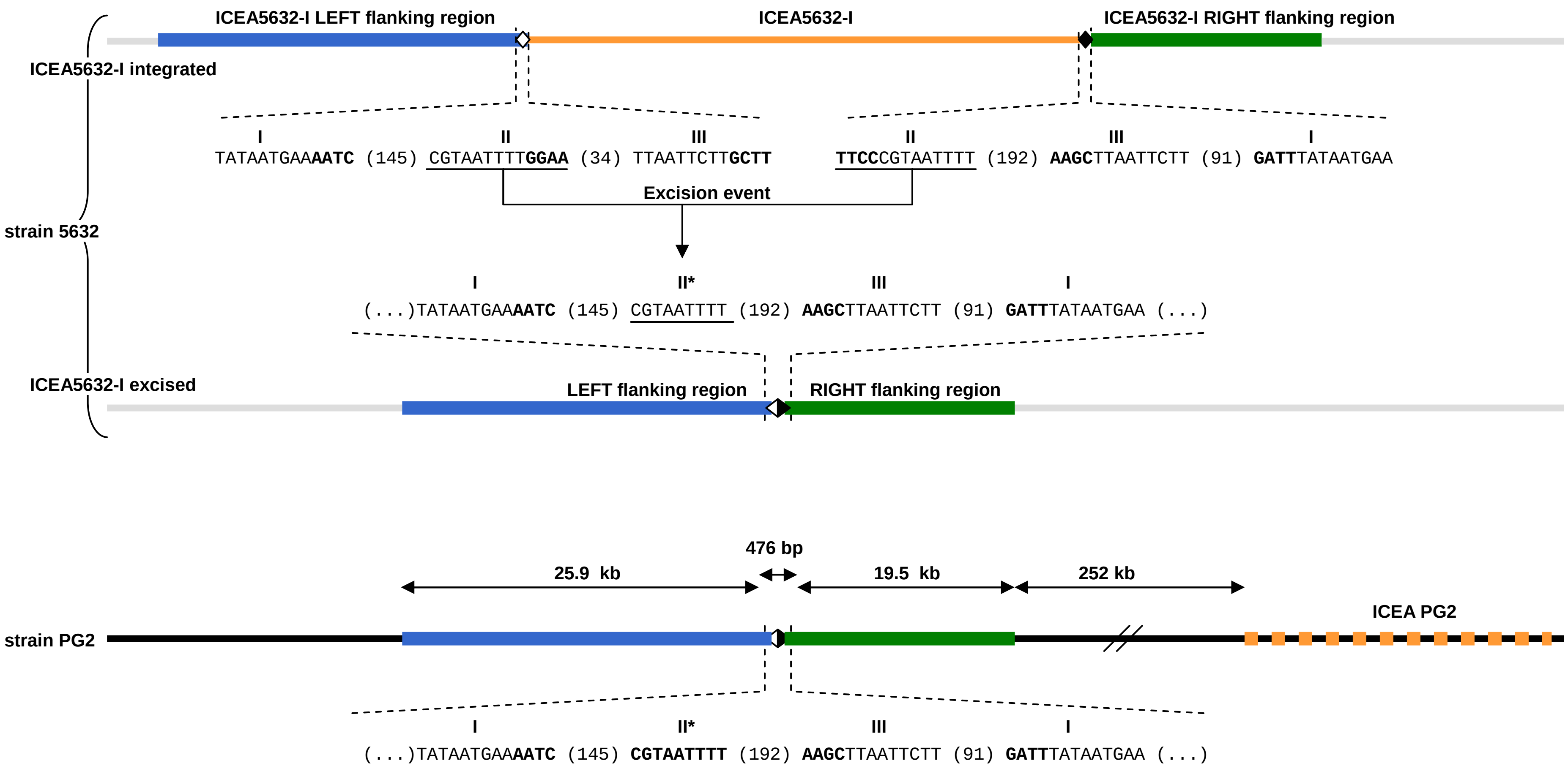

Fig. S7. Alignment of the ICEA5632 locus from the *M. agalactiae* strain 5632 with a synthenic region from *M. agalactiae* PG2. The thick coloured lines represent related sequences whereas grey and blak lines represent non-related regions in 5632 and PG2 strains, respectively. Upper pannel: the blue and green thick lines represent the large regions that flank the integrated copy of ICEA5632-I on the chromosome. The left and right end-sequences of ICEA5632-I are underlined in the close-up text; see Marenda et al (2006) for details. Middle pannel: ICEA5632 locus after the element excision, showing the re-organised end sequences. Lower pannel: PG2 region synthenic to the reorganised ICEA5632 locus. The close-up text indicates the 476 bp sequence that is nearly identical to the ICEA5632 locus after excision of the element in strain 5632. The blue and green thick lines indicate the synthenic region between 5632 and PG2. Dashed orange line represents the ICEAPG2 locus (not to scale). The size of the different regions is indicated above the corresponding double arrows.
