# Supplementary material for: Being Pathogenic, Plastic, and Sexual while Living with a Nearly Minimal Bacterial Genome
Source: PLoS Genet. 2007 May 18;3(5):e75. doi: 10.1371/journal.pgen.0030075 (PMC1868952; doi:10.1371/journal.pgen.0030075)
Supplement: Supplementary file 8 [file pgen.0030075.st001.doc]

**Table S1: Common hosts and tissue tropisms [1,2] of *Mycoplasma,* *Ureaplasma* species and phytoplasmas from which genome has been sequenced**

| Host | Tissue tropisms ^a^ | Mycoplasma species ^b^ |
| --- | --- | --- |
| Human | Respiratory tract | *M. pneumoniae*^d^ |
|  | Urogenital tract | *M. penetrans*^d^  *M. genitalium*^d^  *U. urealyticum/parvum* |
| Birds | Respiratory tract | *M. gallisepticum*^d^  *M. synoviae* |
|  | Joint | *M. gallisepticum*^d^  *M. synoviae* |
|  | Uro-genital tract | *M. gallisepticum*^d^ |
| Ruminants | Respiratory tract | Mycoides cluster ^c^  *M. agalactiae* |
|  | Joint | Mycoides cluster ^c^  *M. agalactiae* |
|  | Mammary gland | Mycoides cluster ^c^  *M. agalactiae* |
| Swine | Respiratory tract | *M. hyopneumoniae* |
| Fish | Gills | *M. mobile*^e^ |
| Plants | Vascular system (phloem sap) | Phytoplasmas |
| Insects | Intestinal tract, haemolymph and salivary glands |  |

^a^ Organs from which mycoplasma species are commonly isolated

^b^ Mycoplasma and ureaplasma species which genome has been sequenced and published

^c^ See Fig. 1 for the mollicutes included in the mycoides cluster from which *M. mycoides* subsp*. mycoides* SC *and M. capricolum* subsp*. capricolum* have been sequenced

^d^ Shown to occur as facultative intracellular

^e^ Only isolated once

1. Razin S, Yogev D, Naot Y (1998) Molecular biology and pathogenicity of mycoplasmas. Microbiol Mol Biol Rev 62: 1094-1156.

2. Rosengarten R, Citti C, Much P, Spergser J, Droesse M, et al. (2001) The changing image of mycoplasmas: from innocent bystanders to emerging and reemerging pathogens in human and animal diseases. Contrib Microbiol 8: 166-185.
