# Supplementary material for: Being Pathogenic, Plastic, and Sexual while Living with a Nearly Minimal Bacterial Genome
Source: PLoS Genet. 2007 May 18;3(5):e75. doi: 10.1371/journal.pgen.0030075 (PMC1868952; doi:10.1371/journal.pgen.0030075)
Supplement: Supplementary file 9 [file pgen.0030075.st002.doc]

**Table S2.** **CDS candidates for HGT in between *M. agalactiae* (MAG) and mycoplasmas of the mycoides cluster**, ***M. mycoides subsp. mycoides* (MmSC) *and M. capricolum subsp. capricolum* (MCAP)**

| Mnemonic | Product | Homolog in MmmSC | Homolog in MCAP | Bootstrap test^a^ (%) | Comment |
| --- | --- | --- | --- | --- | --- |
| **MAG CDS having no homolog outside of the mycoides cluster** | | | | | |
| MAG0190 | Conserved hypothetical protein, truncated in N- and C-terminal | MSC_0345 | MCAP0328 | -^b^ | No homolog outside of the mycoides cluster |
| MAG0200 | Conserved hypothetical protein | MSC_0281 | MCAP0329 | - | No homolog outside of the mycoides cluster |
| MAG0210 | Conserved hypothetical protein, DUF285 family | MSC_0653 | MCAP0287 | - | No homolog outside of the mycoides cluster |
| MAG0220 | Conserved hypothetical protein, truncated in N- and C-terminal | MSC_1005 | MCAP0704 | - | No homolog outside of the mycoides cluster |
| MAG0230 | Conserved hypothetical protein, DUF285 family | MSC_0653 | MCAP0704 | - | No homolog outside of the mycoides cluster |
| MAG1330 | Conserved hypothetical protein, DUF285 family | MSC_1021 | MCAP0300 | - | No homolog outside of the mycoides cluster |
| MAG1340 | Conserved hypothetical protein, DUF285 family | MSC_1021 | MCAP0300 | - | No homolog outside of the mycoides cluster |
| MAG1550 | Conserved hypothetical protein, predicted lipoprotein | NO | MCAP0561 | - | No homolog outside of the mycoides cluster |
| MAG1640 | Conserved hypothetical protein predicted membrane protein | NO | MCAP0440 | - | No homolog outside of the mycoides cluster |
| MAG1670 | Conserved hypothetical protein, predicted lipoprotein | MSC_0240 | MCAP0033 | - | No homolog outside of the mycoides cluster |
| MAG2170 | Conserved hypothetical protein, truncated in N-terminal | MSC_0092 | NO | - | No homolog outside of the mycoides cluster |
| MAG2430 | Conserved hypothetical protein, predicted lipoprotein, DUF285 family | MSC_1005 | MCAP0268 | - | No homolog outside of the mycoides cluster |
| MAG2490 | Conserved hypothetical protein | MSC_0107 | MCAP0329 | - | No homolog outside of the mycoides cluster |
| MAG2610 | Conserved hypothetical protein, predicted lipoprotein | MSC_0281 | MCAP0240 | - | No homolog outside of the mycoides cluster |
| MAG2720 | Conserved hypothetical protein | MSC_0084 | MCAP0820 | - | No homolog outside of the mycoides cluster |
| MAG3260 | Conserved hypothetical protein, DUF285 family | MSC_0418 | MCAP0770 | - | No homolog outside of the mycoides cluster |
| MAG3270 | Conserved hypothetical protein, DUF285 family | MSC_0419 | MCAP0622 | - | No homolog outside of the mycoides cluster |
| MAG3300 | Conserved hypothetical protein | MSC_0779 | MCAP0726 | - | No homolog outside of the mycoides cluster |
| MAG3340 | Conserved hypothetical protein, truncated in N- and C-terminal | MSC_0036 | NO | - | No homolog outside of the mycoides cluster |
| MAG3360 | Conserved hypothetical protein, truncated in C-terminal | NO | MCAP0571 | - | No homolog outside of the mycoides cluster |
| MAG3370 | Conserved hypothetical protein, truncated in C-terminal | NO | MCAP0568 | - | No homolog outside of the mycoides cluster |
| MAG3380 | Conserved hypothetical protein, potentially truncated inC-terminal | NO | MCAP0568 | - | No homolog outside of the mycoides cluster, truncated paralog of MAG3370 |
| MAG3400 | Conserved hypothetical protein, truncated in C-terminal | MSC_0547 | MCAP0425 | - | No homolog outside of the mycoides cluster |
| MAG3410 | Transposase | MSC_1056 | NO | - | No homolog outside of the mycoides cluster |
| MAG3870 | Conserved hypothetical protein | NO | MCAP0568 | - | Part of ICEA, no homolog outside of the mycoides cluster |
| MAG3960 | Conserved hypothetical protein, truncated in N-terminal | NO | MCAP0561 | - | Part of ICEA, no homolog outside of the mycoides cluster |
| MAG3970 | Conserved hypothetical protein, predicted lipoprotein, truncated in C-ter | NO | MCAP0561 | - | Part of ICEA, no homolog outside of the mycoides cluster |
| MAG4060 | Conserved hypothetical protein, truncated in C-terminal | NO | MCAP0555 | - | Part of ICEA, no homolog outside of the mycoides cluster |
| MAG4100 | Pseudogene of Transposase (N-terminal part) | MSC_0571 | NO | - | No homolog outside of the mycoides cluster |
| MAG4110 | Pseudogene of Transposase (C-terminal part) | MSC_0230 | NO | - | No homolog outside of the mycoides cluster |
| MAG4220 | Conserved hypothetical protein, DUF285 family | MSC_0209 | MCAP0292 | - | No homolog outside of the mycoides cluster |
| MAG4230 | Conserved hypothetical protein, truncated in N-terminal | MSC_0998 | MCAP0843 | - | No homolog outside of the mycoides cluster |
| MAG4240 | Conserved hypothetical protein, predicted lipoprotein, truncated in C-ter | MSC_0997 | MCAP0843 | - | No homolog outside of the mycoides cluster |
| MAG4310 | Conserved hypothetical protein, DUF285 family | MSC_1005 | MCAP721 | - | No homolog outside of the mycoides cluster |
| MAG4860 | Pseudogene of Polysaccharide synthase (N-terminal part) | NO | MCAP0063 | - | No homolog outside of the mycoides cluster |
| MAG4870 | Pseudogene of Polysaccharide synthase(C-terminal part) | NO | MCAP0063 | - | No homolog outside of the mycoides cluster |
| MAG5760 | Conserved hypothetical protein | MSC_0107 | MCAP0328 | - | No homolog outside of the mycoides cluster |
| MAG5770 | Conserved hypothetical protein, predicted lipoprotein | MSC_0345 | MCAP0329 | - | No homolog outside of the mycoides cluster |
| MAG6040 | Conserved hypothetical protein, truncated in N-terminal | NO | MCAP0042 | - | No homolog outside of the mycoides cluster |
| MAG6150 | Pseudogene of Transposase (N-terminal part) | MSC_0230 | NO | - | No homolog outside of the mycoides cluster |
| MAG6160 | Pseudogene of Transposase (C-terminal part) | MSC_0230 | NO | - | No homolog outside of the mycoides cluster |
| MAG6410 | Conserved hypothetical protein | MSC_0997 | MCAP0843 | - | No homolog outside of the mycoides cluster |
| MAG6450 | Conserved hypothetical protein | MSC_0477 | MCAP0494 | - | No homolog outside of the mycoides cluster |
| MAG6460 | Conserved hypothetical protein, predicted lipoprotein, DUF285 family | MSC_1005 | MCAP0721 | - | No homolog outside of the mycoides cluster |
| MAG6470 | Pseudogene of Conserved hypothetical protein (C-terminal part) | MSC_0192 | NO | - | No homolog outside of the mycoides cluster |
| MAG6480 | Conserved hypothetical protein, predicted lipoprotein, DUF285 family | MSC_0209 | MCAP0287 | - | No homolog outside of the mycoides cluster |
| MAG6490 | Conserved hypothetical protein, DUF285 family | MSC_1021 | MCAP0300 | - | No homolog outside of the mycoides cluster |
| MAG6570 | Conserved hypothetical protein | MSC_0550 | MCAP0638 | - | No homolog outside of the mycoides cluster |
| MAG7130 | Conserved hypothetical protein, truncated in N-terminal | MSC_0240 | MCAP0034 | - | No homolog outside of the mycoides cluster |
| MAG7270 | Conserved hypothetical protein, predicted lipoprotein, DUF285 family | MSC_1005 | MCAP0721 | - | No homolog outside of the mycoides cluster |
|  |  |  |  |  |  |
| **MAG having homolog in the mycoides cluster and other bacteria but not in the Hominis group** | | | | | |
| MAG0640 | Aspartate-ammonia ligase (AsnA) | MSC_1040 | MCAP0817 | 99 | Homolog in *U.urealyticum, M.penetrans, M.galliseptic.* |
| MAG1490 | D-lactate dehydrogenase | MSC_0034 | MCAP0460 | ns^e^ | Homolog in *M. penetrans* also highly similar |
| MAG2470 | Proton/glutamate symporter | MSC_1018 | MCAP0857 | 100 | Homolog in non mollicute bacteria |
| MAG2960 | Conserved hypothetical protein, predicted lipoprotein | MSC_0897 | MCAP0072 | 100 | Homolog in *U. urealyticum* |
| MAG3680 | Endopeptidase O | MSC_0696 | MCAP0466 | 100 | Homolog in non mollicute bacteria |
| MAG4030 | Conserved hypothetical protein | MSC_0780 | MCAP0556 | ns^f^ | Part of ICEA, homolog in bacteriophages |
| MAG4120 | Oxidoreductase, potentially truncated in N-terminal | MSC_0301 | MCAP0256 | 100 | Homolog in non mollicute bacteria |
| MAG4130 | Conserved hypothetical protein | MSC_0052 | MCAP0846 | 100 | Homolog in *U. urealyticum* |
| MAG4890 | Malate permease | MSC_0035 | MCAP0780 | ns^e^ | Homolog in *M. penetrans* |
| MAG5660 | Conserved hypothetical protein | MSC_0233 | MCAP0196 | 96 | Homolog in *M. penetrans* and non mollicute bacteria |
| MAG5670 | Conserved hypothetical protein, truncated in N-terminal | MSC_0243 | MCAP0196 | 99 | Homolog in *M. penetrans* |
| MAG5750 | 5-formyltetrahydrofolate cyclo-ligase | MSC_0503 | MCAP0467 | 99 | Homolog in *M. penetrans, Me. florum, M. gallisepticum* |
| MAG6440 | Putative prophage protein (ps3) | MSC_0194 | MCAP0493 | 99 | Homolog in *U. urealyticum* |
|  |  |  |  |  |  |
| **MAG having homolog in the Hominis group** | | | | | |
| MAG0030 | Esterase/lipase | MSC_0530 | MCAP0446 | 93 | Several homologs from the Hominis group |
| MAG0040 | Esterase/lipase | MSC_0531 | MCAP0445 | 93 | Several homologs from the Hominis group |
| MAG0050 | NADH dependent flavin oxidoreductase | MSC_0526 | MCAP0450 | 100 | Homolog from the Hominis group probably lost |
| MAG0060 | Lipoate-protein ligase A, truncated in C-terminal | MSC_0527 | MCAP0449 | 99 | Homolog from the Hominis group remaining |
| MAG0070 | Lipoate-protein ligase A | MSC_0527 | MCAP0449 | 100 | Homolog from the Hominis group remaining |
| MAG0080 | Conserved hypothetical protein | MSC_0528 | MCAP0448 | ns^f^ | Homolog from the Hominis group probably lost |
| MAG0090 | Glycine cleavage system H protein | MSC_0529 | MCAP0447 | 100 | Paralog MAG3700 (also acquired from the mycoides group), homolog from the Hominis group probably lost |
| MAG0340 | Oligopeptide ABC transporter, ATP-binding protein (OppF) | MSC_0968 | MCAP0120 | 98 | Homolog from the Hominis group remaining |
| MAG0350 | Oligopeptide ABC transporter, ATP-binding protein (OppD) | MSC_0967 | MCAP0119 | 100 | Homolog from the Hominis group remaining |
| MAG0360 | Oligopeptide ABC transporter, permease protein(OppC) | MSC_0966 | MCAP0118 | 100 | Homolog from the Hominis group remaining |
| MAG0370 | Oligopeptide ABC transporter, permease protein(OppB) | MSC_0965 | MCAP0117 | 100 | Homolog from the Hominis group remaining |
| MAG0380 | Oligopeptide ABC transporter, substrate-binding protein (OppA), predicted lipoprotein | MSC_0964 | MCAP0116 | 100 | Several homologs from the Hominis group |
| MAG0390 | Conserved hypothetical protein | MSC_0963 | MCAP0115 | 100 | Homolog from the Hominis group remaining |
| MAG1470 | Glucose-inhibited division protein | MSC_0375 | MCAP0613 | 100 | Homolog from the Hominis group remaining |
| MAG1500 | Esterase/lipase | MSC_0368 | MCAP0606 | 93 | Homolog from the Hominis group remaining |
| MAG2150 | Pseudogene of Exodeoxyribonuclease V alphachain (RecD) (C-terminal part) | MSC_0512 | MCAP0455 | 100^c^ | Homolog from the Hominis group remaining |
| MAG2160 | Pseudogene of Exodeoxyribonuclease V alphachain (RecD) (N-terminal part) | MSC_0512 | MCAP0456 |  | Homolog from the Hominis group remaining |
| MAG2220 | Conserved hypothetical protein, predicted lipoprotein | MSC_0519 | MCAP0451 | 100 | Several homologs |
| MAG2300 | Esterase/lipase | MSC_0530 | MCAP0446 | 93 | Homolog from the Hominis group remaining |
| MAG2310 | Glycerol ABC transporter, ATP-binding component | MSC_0516 | MCAP0454 | 100 | Several homologs |
| MAG2320 | Glycerol ABC transporter, permease component | MSC_0517 | MCAP0453 | 100 | Several homologs |
| MAG2330 | Glycerol ABC transporter, permease component | MSC_0518 | MCAP0452 | 100 | Several homologs |
| MAG2340 | Conserved hypothetical protein | MSC_0519 | MCAP0451 | 100 | Several homologs |
| MAG2460 | NADH oxidase (NOXASE) | MSC_1019 | MCAP0858 | 100 | Homolog from the Hominis group remaining |
| MAG2690 | Alkylphosphonate ABC transporter,substrate-binding protein, predicted lipoprotein | MSC_0790 | MCAP0731 | 100 | Homolog from the Hominis group probably lost |
| MAG2700 | ABC transporter, ATP-binding protein | MSC_0789 | MCAP0730 | 100 | Several homologs from the Hominis group |
| MAG2710 | Alkylphosphonate ABC transporter, permease protein | MSC_0788 | MCAP0729 | 100 | Homolog from the Hominis group probably lost |
| MAG2830 | Conserved hypothetical protein, predicted transmembrane protein | MSC_0626 | MCAP0349 | 100 | Homolog from the Hominis group probably lost |
| MAG2840 | Conserved hypothetical protein, predicted lipoprotein | MSC_0635 | MCAP0350 | 100 | Paralog MAG2870, 3120 (also acquired from the mycoides group) |
| MAG2850 | Conserved hypothetical protein, predicted transmembrane protein | MSC_0628 | MCAP0349 | 100 | Homolog from the Hominis group probably lost |
| MAG2860 | Conserved hypothetical protein, predicted transmembrane protein | MSC_0626 | MCAP0351 | 100 | Homolog from the Hominis group probably lost |
| MAG2870 | Conserved hypothetical protein, predicted lipoprotein | MSC_0625 | MCAP0352 | 100 | Paralog MAG2840, 3120 (also acquired from the mycoides group) |
| MAG2880 | Conserved hypothetical protein, predicted transmembrane protein | MSC_0624 | MCAP0353 | 100 | Homolog from the Hominis group probably lost |
| MAG2890 | Conserved hypothetical protein | MSC_0623 | MCAP0354 | 70^d^ | Homolog from the Hominis group probably lost |
| MAG2900 | Conserved hypothetical protein | MSC_0622 | MCAP0355 | 96 | Homolog from the Hominis group probably lost |
| MAG2910 | Conserved hypothetical protein | MSC_0621 | MCAP0356 | 73 ^d^ | No paralog |
| MAG2920 | Conserved hypothetical protein, predicted transmembrane protein | MSC_0620 | MCAP0357 | 100 | Homolog from the Hominis group probably lost |
| MAG2930 | ATP synthase alpha chain | MSC_0619 | MCAP0358 | 100 | Homolog from the Hominis group remaining |
| MAG2940 | ATP synthase beta chain | MSC_0618 | MCAP0359 | 100 | Homolog from the Hominis group remaining |
| MAG3120 | Conserved hypothetical protein, predicted lipoprotein | MSC_0271 | MCAP0231 | 100 | Paralog MAG2840, 2870 (also acquired from the mycoides group) |
| MAG3130 | Peptide methionine sulfoxide reductase | MSC_0774 | MCAP0722 | 100 | Homolog from the Hominis group probably lost |
| MAG3290 | Conserved hypothetical protein | MSC_0778 | MCAP0725 | 100 | No paralog |
| MAG3330 | Conserved hypothetical protein | MSC_0037 | NO | ns^f^ | Homolog in *M. penetrans, M. fermentans* |
| MAG3700 | Glycine cleavage system H protein | MSC_0529 | MCAP0447 | 99 | Paralog MAG0090 (also acquired from the mycoides group), homolog from the Hominis group probably lost |
| MAG3860 | Conserved hypothetical protein | NO | MCAP0571 | -^b^ | Part of ICEA, only a weak similarity with *M. fermentans* ICEF |
| MAG3880 | Pseudogen of conserved hypothetical protein (C-terminal part) | NO | MCAP0567 | 100 | Part of ICEA, homolog in *M. fermentans, M. hyopneumoniae* |
| MAG3890 | Pseudogen of conserved hypothetical protein (N-terminal part) | NO | MCAP0567 | 100 | Part of ICEA, homolog in *M. fermentans, M. hyopneumoniae* |
| MAG3900 | Conserved hypothetical protein | NO | MCAP0566 | 100 | Part of ICEA, homolog in *M. fermentans, M. hyopneumoniae* |
| MAG3910 | Pseudogene of TraE/TrsE family NTPase(C-terminal part) | NO | MCAP0565 | 100c | Part of ICEA, homolog in *M. fermentans, M. hyopneumoniae* |
| MAG3920 | Pseudogene of TraE/TrsE family NTPase(N-terminal part) | NO | MCAP0566 |  | Part of ICEA, homolog in *M. fermentans, M. hyopneumoniae* |
| MAG3930 | Pseudogen of conserved hypothetical protein (C-terminal part) | NO | MCAP0564 | -^b^ | Part of ICEA, only a weak similarity with *M. fermentans* ICEF |
| MAG3940 | Pseudogen of conserved hypothetical protein (N-terminal part) | NO | MCAP0564 | -^b^ | part of ICEA, only a weak similarity with *M. fermentans* ICEF |
| MAG4040 | Conserved hypothetical protein | NO | MCAP0559 | 100 | Part of ICEA, homolog in *M. fermentans, M. hyopneumoniae* |
| MAG4050 | Conserved hypothetical protein | NO | MCAP0555^g^ | ns^f^ | Part of ICEA, homolog in *M. fermentans, M. hyopneumoniae* |
| MAG4270 | Conserved hypothetical protein | NO | NO | 100 | Similar to Hypothetical protein of Mycoplasma mycoides subsp. capri (tr\|Q9RQS4) |
| MAG4470 | Glycerol kinase | MSC_0258 | MCAP0218 | 100 | Homolog from the Hominis group probably lost |
| MAG4480 | Glycerol facilitator factor | MSC_0257 | MCAP0217 | 100 | Homolog from the Hominis group probably lost |
| MAG4600 | ABC transporter, permease protein | MSC_0324 | MCAP0798 | 100 | No paralog |
| MAG4610 | ABC transporter, ATP-binding protein | MSC_0323 | MCAP0797 | 99 | Several homologs |
| MAG4620 | Conserved hypothetical protein | MSC_0322 | MCAP0796 | 99 | No paralog |
| MAG4630 | Conserved hypothetical protein | MSC_0321 | MCAP0795 | 100 | No paralog |
| MAG4640 | Conserved hypothetical protein, predicted lipoprotein | MSC_0320 | MCAP0794 | 98 | No paralog |
| MAG4840 | Pseudogene of Triacylglycerol lipase (N-terminal part) | MSC_0531 | MCAP0445 | 100 ^c^ | Homolog from the Hominis group remaining |
| MAG4850 | Pseudogene of Triacylglycerol lipase (C-terminal part) | MSC_0531 | MCAP0446 |  | Homolog from the Hominis group remaining |
| MAG4940 | Transmembrane protein | MSC_0777 | MCAP0724 | 100 | No paralog |
| MAG4950 | Pseudogene of Lipoprotein (N-terminal part) | MSC_0775 | MCAP0723 | 100 ^c^ | No paralog |
| MAG4960 | Pseudogene of Lipoprotein (C-terminal part) | MSC_0775 | MCAP0723 |  | No paralog |
| MAG4970 | Hexosephosphate transport protein | MSC_0118 | MCAP0814 | 100 | Homolog from the Hominis group probably lost |
| MAG6520 | Conserved hypothetical protein, predicted lipoprotein | MSC_0519 | MCAP0451 | 100 | Several homologs |
| MAG6530 | Glycerol ABC transporter, permease component | MSC_0518 | MCAP0452 | 100 | Several homologs |
| MAG6540 | Glycerol transporter subunit B | MSC_0517 | MCAP0453 | 100 | Several homologs |
| MAG6550 | Pseudogene of Glycerol transporter subunit A (C-terminal part) | MSC_0516 | MCAP0454 | 100^c^ | Several homologs |
| MAG6560 | Pseudogene of Glycerol transporter subunit A (N-terminal part) | MSC_0516 | MCAP0454 |  | Several homologs |

^a^ bootstrap value supporting the association of *M. agalactiae* protein with homologs from the mycoides cluster in a phylogenetic reconstruction using the neighbour-joining method (bootstrap test with 500 replicates)

^b^ bootstrap test non applicable since homologs were only found in the mycoides cluster

^c^ both part of the pseudogen were fused for phylogenetic reconstruction

^d^ this gene was included in the list because (i) best blast hits were found in species from the mycoides cluster, (ii) this gene is part of a group of putative xenologs which synteny is conserved in species from the mycoides cluster.

^e^ Best BLAST hit in species from the mycoides cluster but non significant bootstrap value

^f^ non significant bootstrap value. This gene was included in the list because (i) best blast hits were found in species from the mycoides cluster, (ii) this gene is part of a group which synteny is conserved in species from the mycoides cluster.

^g^ In the original annotation MCAP0555 was not defined in the correct reading frame.
