# Supplementary material for: Being Pathogenic, Plastic, and Sexual while Living with a Nearly Minimal Bacterial Genome
Source: PLoS Genet. 2007 May 18;3(5):e75. doi: 10.1371/journal.pgen.0030075 (PMC1868952; doi:10.1371/journal.pgen.0030075)
Supplement: Supplementary file 10 [file pgen.0030075.st003.doc]

**Table S3. CDS candidates for HGT in between *M. agalactiae* and mycoplasmas of the Pneumoniae group or non-mollicute bacteria**.

| Mnemonic | Product | Putative HGT with organisms of | Bootstrap test^c^ (%) | Comment |
| --- | --- | --- | --- | --- |
| MAG1070 | Pseudogene of CHP^a^ (N-terminal part) | Pneumoniae group | - ^d^ | No homolog in the Hominis group |
| MAG1080 | Pseudogene of CHP (C-terminal part) | Pneumoniae group | - | No homolog in the Hominis group |
| MAG4460 | CHP^a^, predicted lipoprotein | Pneumoniae group | - | No homolog in the Hominis group, homolog only in *U. urealyticum* |
| MAG4510 | CHP | Pneumoniae group | 99 | Paralog from the Hominis group probably lost |
| MAG0250 | CHP | Firmicutes/Proteobacteria | - | No paralog, no homolog in *Mollicutes* |
| MAG0260 | CHP | Firmicutes/Proteobacteria | - | No paralog, no homolog in *Mollicutes* |
| MAG1230 | Phosphoketolase | Firmicutes (Lactobacillales) | - | No paralog, no homolog in *Mollicutes* |
| MAG2740 | Alcohol dehydrogenase | Firmicutes/Proteobacteria | 100 | Paralog MAG4280, MAG4340 (also HGT acquired), no homolog in the Hominis group |
| MAG4280 | Alcohol dehydrogenase | Firmicutes/Proteobacteria | 99 | Paralog MAG2740, 4340 (also HGT acquired), no homolog in the Hominis group |
| MAG4340 | Alcohol dehydrogenase | Firmicutes/Proteobacteria | 99 | Paralog MAG4280, MAG2740 (also HGT acquired), no homolog in the Hominis group |
| MAG5650 | Methylase of type I RM^b^ system | Firmicutes | 98 | Paralog MAG5730 (also HGT acquired), paralog from the Hominis group probably lost |
| MAG5690 | Phage family integrase | Proteobacteria/ Bacilli | - | No paralog, no homolog in *Mollicutes* |
| MAG5730 | Methylase of type I RM^b^ system HsdM | Firmicutes | 95 | Paralog MAG5730 (also HGT acquired), paralog from the Hominis group probably lost |
| MAG6680 | Modification methylase | Proteobacteria/ Lactobacillales | 99 | No paralog, homolog only in MmmSC |

^a^CHP, conserved hypothetical protein

^b^RM, restriction/modification

^c^ bootstrap value supporting the association of *M. agalactiae* protein with homologs from the putative origin in a phylogenetic reconstruction using the neighbour-joining method (bootstrap test with 500 replicates)

^d^ bootstrap test non applicable since homologs were only found in the putative origin
