# Supplementary material for: Being Pathogenic, Plastic, and Sexual while Living with a Nearly Minimal Bacterial Genome
Source: PLoS Genet. 2007 May 18;3(5):e75. doi: 10.1371/journal.pgen.0030075 (PMC1868952; doi:10.1371/journal.pgen.0030075)
Supplement: Supplementary file 11 [file pgen.0030075.st004.doc]

**Table S4: Fastap alignments of ICEA_PG2_ CDS products with their homologs in ICEC, ICEA_5632_-I and ICEF.**

| ICEA_PG2_ (*M. agalactiae* PG2)  mnemonic | ICEC (*M. capricolum* california kid) | | | | ICEA_5632_-I (*M. agalactiae* 5632) | | | | ICEF (*M. fermentans* PG18) | | | |
| --- | --- | --- | --- | --- | --- | --- | --- | --- | --- | --- | --- | --- |
|  | mnemonic | % identity | % similarity | aa overlap | CDS^a^ | % identity | % similarity | aa overlap | ORF^b^ | % identity | % similarity | aa overlap |
| MAG4060 | MCAP0555 | 47 | 79 | 53 | absent | na | na | na | 3 | 22 | 56 | 105 |
| MAG4050 | MCAP0555 | 37 | 70 | 323 | absent | na | na | na | 3 | 25 | 62 | 269 |
| MAG4040 | MCAP0559 | 52 | 80 | 594 | 5 | 30 | 66 | 603 | 5 | 34 | 69 | 563 |
| MAG4030 | MCAP0556 | 47 | 72 | 188 | absent | na | na | na | absent | na | na | na |
| MAG3990 | absent | na | na | na | 12 | 33 | 70 | 112 | 12 | 30 | 70 | 96 |
| MAG3980 | absent | na | na | na | absent | na | na | na | 11 | 27 | 60 | 177 |
| MAG3970 | MCAP0561 | 30 | 64 | 320 | absent | na | na | na | absent | na | na | na |
| MAG3960 | MCAP0561 | 41 | 71 | 121 | absent | na | na | na | absent | na | na | na |
| MAG3940 | MCAP0564 | 40 | 79 | 192 | 16 | 22 | 69 | 129 | 16 | 26 | 70 | 130 |
| MAG3930 | MCAP0564 | 30 | 66 | 386 | 16 | 20 | 61 | 125 | 16 | 28 | 62 | 157 |
| MAG3920 | MCAP0565 | 55 | 89 | 102 | absent | na | na | na | 17 | 28 | 62 | 108 |
| MAG3910 | MCAP0565 | 64 | 87 | 704 | 17 | 29 | 66 | 704 | 17 | 31 | 65 | 717 |
| MAG3900 | MCAP0566 | 42 | 78 | 186 | absent | na | na | na | 18 | 29 | 68 | 192 |
| MAG3890 | MCAP0567 | 39 | 65 | 839 | 19 | 25 | 53 | 722 | 19 | 28 | 58 | 519 |
| MAG3890 | MCAP0567 | 34 | 60 | 535 | 19 | 23 | 52 | 406 | absent | na | na | na |
| MAG3870 | MCAP0568 | 49 | 78 | 201 | absent | na | na | na | absent | na | na | na |
| MAG3860 | MCAP0571 | 52 | 83 | 406 | 22 | 25 | 59 | 388 | 22 | 24 | 57 | 384 |

a: CDS number as in ref. [1]

b: ORF number as in ref. [2]

Percentages of identity (normal font) and similarity (bold font) between conserved ICE sequences from *M. agalactiae* PG2 (this study, MA PG2), *M. capricolum* california kid (acc. NC_007633, MC kid), *M. agalactiae* 5632 (MA 5632) *M. fermentans* PG18 (MF PG18), and *M. hyopneumoniae* 7448 [3] (MH 7448).

|  | ICE ORF5 sequence^a^ | | | | | ICE ORF17 sequence^b^ | | | | | ICE ORF22 sequence^c^ | | | | |
| --- | --- | --- | --- | --- | --- | --- | --- | --- | --- | --- | --- | --- | --- | --- | --- |
|  | MA PG2 | MC kid | MA 5632 | MF PG18 | MH 7448 | MA PG2 | MC kid | MA 5632 | MF PG18 | MH 7448 | MA PG2 | MC kid | MA 5632 | MF PG18 | MH 7448 |
| MA PG2 | 100 | 52 | 30 | 34 | 28 | 100 | 64 | 29 | 31 | 32 | 100 | 52 | 26 | 24 | 25 |
| MC kid | **80** | 100 | 31 | 30 | 27 | **87** | 100 | 28 | 29 | 30 | **83** | 100 | 27 | 24 | 28 |
| MA 5632 | **66** | **63** | 100 | 48 | 26 | **66** | **63** | 100 | 57 | 25 | **59** | **58** | 100 | 41 | 28 |
| MF PG18 | **69** | **65** | **77** | 100 | 26 | **65** | **61** | **85** | 100 | 27 | **57** | **58** | **67** | 100 | 25 |
| MH 7448 | **64** | **58** | **58** | **58** | 100 | **69** | **65** | **61** | **62** | 100 | **61** | **58** | **60** | **58** | 100 |

a: MAG4040, MCAP0559, CDS5, ORF5, MHP7448_423

b: MAG3910, MCAP0565, CDS17, ORF17, MHP7448_416

c: MAG3860, MCAP0571CDS22, ORF22, MHP7448_412

1.	Marenda, M., et al., *A New Integrative Conjugative Element Occurs in Mycoplasma agalactiae as Chromosomal and Free Circular Forms.* J. Bacteriol., 2006. **188**(11): p. 4137-41.

2.	Calcutt, M.J., M.S. Lewis, and K.S. Wise, *Molecular genetic analysis of ICEF, an integrative conjugal element that is present as a repetitive sequence in the chromosome of Mycoplasma fermentans PG18.* J. Bacteriol., 2002. **184**(24): p. 6929-41.

3.	Vasconcelos, A.T., et al., *Swine and poultry pathogens: the complete genome sequences of two strains of Mycoplasma hyopneumoniae and a strain of Mycoplasma synoviae.* J. Bacteriol., 2005. **187**(16): p. 5568-77.
