# Supplementary material for: Being Pathogenic, Plastic, and Sexual while Living with a Nearly Minimal Bacterial Genome
Source: PLoS Genet. 2007 May 18;3(5):e75. doi: 10.1371/journal.pgen.0030075 (PMC1868952; doi:10.1371/journal.pgen.0030075)
Supplement: Supplementary file 12 [file pgen.0030075.st005.doc]

**Table S5: CDS candidates for HGT in between *M. mycoides subsp. mycoides SC* and *M. agalactiae* (MAG)**

| Mnemonic | Product | BBH^a^ | Bootstrap values^b^ (%) | Comments |
| --- | --- | --- | --- | --- |
| **MSC CDS having homologs only in the Hominis group** | |  |  |  |
| MSC_0092 | Hypothetical protein | MAG2170 | -^c^ | No other homolog |
| MSC_0037 | Conserved hypothetical protein | MAG3330 | - | No other homolog |
| MSC_0230 | Transposase ISMmy1B | MAG3410 | - | No other homolog |
| MSC_0808 | TRANSPOSASE ISMmy1C | MAG3410 | - | No other homolog |
| MSC_1031 | Transposase ISMmy1A | MAG3410 | - | No other homolog |
| MSC_1056 | Transposase ISMmy1I | MAG3410 | - | No other homolog |
| MSC_0123 | transposase ISMmy1E | MAG3410 | - | No other homolog |
| MSC_0571 | Transposase ISMmy1F | MAG3410 | - | No other homolog |
| MSC_0796 | TRANSPOSASE ISMmy1G | MAG3410 | - | No other homolog |
| MSC_0781 | Transposase ISMmy1D | MAG3410 | - | No other homolog |
| MSC_0659 | Fic family protein | MAG6470 | - | No other homolog |
| MSC_0192 | Conserved hypothetical protein, Fic protein family | MAG6470 | - | No other homolog |
| MSC_0122 | Prolipoprotein lppC | MAG7270 | - | No other homolog |
| MSC_1061 | hypothetical protein | MGA_0535 | - | No other homolog |
| MSC_1062 | AAA family ATPase | MGA_0536 | - | No other homolog |
| MSC_0516 | Glycerol ABC transporter, ATP-binding component | MAG2310 | 100 | Homolog in several species of the hominis group |
|  |  |  |  |  |
| **MSC CDS having homologs only outside of the Spiroplasma group** | |  |  |  |
| MSC_0245 | Transposase IS1634AC | mhp082 | - | Homologs in the pneumoniae group |
| MSC_0921 | Transposase IS1634CD | mhp082 | - | Homologs in the pneumoniae group |
| MSC_0070 | Transposase IS1634BA | mhp082 | - | Homologs in the pneumoniae group |
| MSC_0697 | Transposase IS1634BM | mhp082 | - | Homologs in the pneumoniae group |
| MSC_0520 | Transposase IS1634AD | mhp082 | - | Homologs in the pneumoniae group |
| MSC_0342 | Transposase IS1634BR | mhp082 | - | Homologs in the pneumoniae group |
| MSC_0551 | Transposase IS1634AA | mhp082 | - | Homologs in the pneumoniae group |
| MSC_0168 | Transposase IS1634BZ | mhp082 | - | Homologs in the pneumoniae group |
| MSC_1003 | Transposase IS1634BG | mhp082 | - | Homologs in the pneumoniae group |
| MSC_0097 | Transposase IS1634BO | mhp082 | - | Homologs in the pneumoniae group |
| MSC_0098 | Transposase IS1634AP | mhp082 | - | Homologs in the pneumoniae group |
| MSC_0785 | Transposase IS1634AM | mhp082 | - | Homologs in the pneumoniae group |
| MSC_0802 | Transposase IS1634AY | mhp082 | - | Homologs in the pneumoniae group |
| MSC_0989 | Transposase IS1634CB | mhp082 | - | Homologs in the pneumoniae group |
| MSC_1011 | Transposase IS1634BX | mhp082 | - | Homologs in the pneumoniae group |
| MSC_1036 | Transposase IS1634CM | mhp082 | - | Homologs in the pneumoniae group |
| MSC_0060 | Transposase IS1634BK | mhp082 | - | Homologs in the pneumoniae group |
| MSC_0074 | Transposase IS1634BV | mhp082 | - | Homologs in the pneumoniae group |
| MSC_0332 | Transposase IS1634AW | mhp082 | - | Homologs in the pneumoniae group |
| MSC_0814 | Transposase IS1634CHBZ | mhp082 | - | Homologs in the pneumoniae group |
| MSC_0934 | Transposase IS1634BY | mhp082 | - | Homologs in the pneumoniae group |
| MSC_0969 | Transposase IS1634BQ | mhp082 | - | Homologs in the pneumoniae group |
| MSC_0981 | Transposase IS1634AV | mhp082 | - | Homologs in the pneumoniae group |
| MSC_0038 | Transposase IS1634BT | mhp082 | - | Homologs in the pneumoniae group |
| MSC_0172 | Transposase IS1634BP | mhp082 | - | Homologs in the pneumoniae group |
| MSC_0249 | Transposase IS1634AG | mhp082 | - | Homologs in the pneumoniae group |
| MSC_0363 | Transposase IS1634AE | mhp082 | - | Homologs in the pneumoniae group |
| MSC_0538 | Transposase IS1634AB | mhp082 | - | Homologs in the pneumoniae group |
| MSC_0602 | Transposase IS1634CA | mhp082 | - | Homologs in the pneumoniae group |
| MSC_0605 | Transposase IS1634BH | mhp082 | - | Homologs in the pneumoniae group |
| MSC_0672 | Transposase IS1634AS | mhp082 | - | Homologs in the pneumoniae group |
| MSC_0685 | Transposase IS1634BL | mhp082 | - | Homologs in the pneumoniae group |
| MSC_0797 | TRANSPOSASE IS1634AU | mhp082 | - | Homologs in the pneumoniae group |
| MSC_0822 | TRANSPOSASE IS1634CI | mhp082 | - | Homologs in the pneumoniae group |
| MSC_0859 | Transposase IS1634AL | mhp082 | - | Homologs in the pneumoniae group |
| MSC_0862 | Transposase IS1634AX | mhp082 | - | Homologs in the pneumoniae group |
| MSC_0871 | Transposase IS1634CE | mhp082 | - | Homologs in the pneumoniae group |
| MSC_0876 | Transposase IS1634AZ | mhp082 | - | Homologs in the pneumoniae group |
| MSC_0976 | Transposase IS1634BN | mhp082 | - | Homologs in the pneumoniae group |
|  |  |  |  |  |
| **MSC CDS having homologs in the Spiroplasma group and in *M. agalactiae* only** | | | |  |
| MSC_0136 | Hypothetical transmembrane protein | MAG4220 | - | No other homolog outside of the spiroplasma group, DUF285 family |
| MSC_0900 | HYPOTHETICAL TRANSMEMBRANE PROTEIN | MAG4220 | - | No other homolog outside of the spiroplasma group, DUF285 family |
| MSC_0208 | Conserved hypothetical protein | MAG1340 | - | No other homolog outside of the spiroplasma group, DUF285 family |
| MSC_0419 | prolipoprotein | MAG3270 | - | No other homolog outside of the spiroplasma group, DUF285 family |
| MSC_0431 | Prolipoprotein | MAG1330 | - | No other homolog outside of the spiroplasma group, DUF285 family |
| MSC_1021 | Prolipoprotein Q | **MAG1340** | - | No other homolog outside of the spiroplasma group, DUF285 family |
| MSC_1046 | Prolipoprotein Q | **MAG1340** | - | No other homolog outside of the spiroplasma group, DUF285 family |
| MSC_0209 | Conserved hypothetical prolipoprotein | MAG3270 | - | No other homolog outside of the spiroplasma group, DUF285 family |
| MSC_1005 | Putative variable surface prolipoprotein | MAG7270 | - | No other homolog outside of the spiroplasma group, DUF285 family |
| MSC_0755 | Conserved hypothetical protein | MAG0230 | - | No other homolog outside of the spiroplasma group, DUF285 family |
| MSC_0177 | Prolipoprotein C | MAG7270 | - | No other homolog outside of the spiroplasma group, DUF285 family |
| MSC_0773 | Conserved hypothetical protein | MAG4310 | - | No other homolog outside of the spiroplasma group, DUF285 family |
| MSC_0812 | putative variable surface protein | MAG3270 | - | No other homolog outside of the spiroplasma group, DUF285 family |
| MSC_0815 | putative variable surface protein | MAG3270 | - | No other homolog outside of the spiroplasma group, DUF285 family |
| MSC_0816 | putative variable surface protein | MAG3270 | - | No other homolog outside of the spiroplasma group, DUF285 family |
| MSC_0901 | HYPOTHETICAL TRANSMEMBRANE PROTEIN | MAG3270 | - | No other homolog outside of the spiroplasma group, DUF285 family |
| MSC_0809 | putative variable surface protein | MAG3270 | - | No other homolog outside of the spiroplasma group, DUF285 family |
| MSC_0818 | putative variable surface protein | MAG3270 | - | No other homolog outside of the spiroplasma group, DUF285 family |
| MSC_0014 | IMMUNODOMI -NT PROTEIN P72 PRECURSOR | **MAG1670** | - | No other homolog outside of the spiroplasma group |
| MSC_0013 | Prolipoprotein | **MAG1670** | - | No other homolog outside of the spiroplasma group |
| MSC_0240 | IMMUNODOMI -NT PROTEIN P72 PRECURSOR | **MAG1670** | - | No other homolog outside of the spiroplasma group |
| MSC_0247 | hypothetical protein | MAG1330 | - | No other homolog outside of the spiroplasma group |
| MSC_0281 | Hypothetical transmembrane protein | MAG5760 | - | No other homolog outside of the spiroplasma group |
| MSC_0669 | Hypothetical protein | MAG6480 | - | No other homolog outside of the spiroplasma group |
| MSC_0107 | Hypothetical transmembrane protein | MAG5760 | - | No other homolog outside of the spiroplasma group |
| MSC_0528 | Conserved hypothetical protein | MAG0080 | - | No other homolog outside of the spiroplasma group |
| MSC_0650 | hypothetical protein | MAG2160 | - | No other homolog outside of the spiroplasma group |
| MSC_0478 | Conserved hypothetical protein | MAG6440 | - | No other homolog outside of the spiroplasma group |
| MSC_0477 | Hypothetical protein | MAG6450 | - | No other homolog outside of the spiroplasma group |
| MSC_0369 | hypothetical protein | MAG4230 | - | No other homolog outside of the spiroplasma group |
| MSC_0362 | hypothetical protein | MAG6570 | - | No other homolog outside of the spiroplasma group |
| MSC_0784 | Hypothetical transmembrane protein | MAG4940 | - | No other homolog outside of the spiroplasma group |
| MSC_0800 | Hypothetical transmembrane protein | MAG4940 | - | No other homolog outside of the spiroplasma group |
| MSC_0777 | Hypothetical transmembrane protein | MAG4940 | - | No other homolog outside of the spiroplasma group |
| MSC_0171 | Hypothetical protein | MAG4230 | - | No other homolog outside of the spiroplasma group |
| MSC_0169 | Conserved hypothetical protein | **MAG6410** | - | No other homolog outside of the spiroplasma group |
| MSC_0170 | Hypothetical protein | **MAG6410** | - | No other homolog outside of the spiroplasma group |
| MSC_0084 | Conserved HYPOTHETICAL PROTEIN | MAG2720 | - | No other homolog outside of the spiroplasma group |
| MSC_0061 | Prolipoprotein | MAG4240 | - | No other homolog outside of the spiroplasma group |
| MSC_0998 | Hypothetical protein | MAG6410 | - | No other homolog outside of the spiroplasma group |
| MSC_0997 | Transglutami -se | MAG6410 | - | No other homolog outside of the spiroplasma group |
| MSC_0052 | Conserved HYPOTHETICAL PROTEIN | MAG4130 | - | No other homolog outside of the spiroplasma group |
| MSC_0343 | hypothetical protein (truncated by IS1634BR) | MAG5760 | - | No other homolog outside of the spiroplasma group |
| MSC_0550 | Conserved hypothetical transmembrane protein | MAG6570 | - | No other homolog outside of the spiroplasma group |
| MSC_1018 | proton/glutamate symporter | MAG2470 | 100 | No other homolog outside of the spiroplasma group |
| MSC_1043 | proton/glutamate symporter | MAG2470 | 100 | No other homolog outside of the spiroplasma group |
| MSC_0345 | HYPOTHETICAL PROTEIN | MAG5770 | 100 | No other homolog outside of the spiroplasma group |
| MSC_0343 | hypothetical protein (truncated by IS1634BR) | MAG5760 | - | No other homolog outside of the spiroplasma group |
|  |  |  |  |  |
| **MSC CDS having homologs in the Spiroplasma group and other mollicutes or bacteria** | | | |  |
| MSC_0504 | peptidase | MAG3680 | 52^d^ | Homologs in several mollicutes and non mollicute bacteria |
| MSC_0920 | Conserved hypothetical protein | MAG4600 | 63 ^d^ | Homologs in the Hominis group |
| MSC_0530 | triacylglycerol lipase | MAG0030 | 68 ^d^ | Homologs in several mollicutes and non mollicute bacteria |
| MSC_0321 | hypothetical transmembrane protein | MAG4630 | 70 ^d^ | Homologs in the Hominis group |
| MSC_0923 | Conserved hypothetical transmembrane protein | MAG4630 | 70 ^d^ | Homologs in the Hominis group |
| MSC_0623 | Conserved hypothetical protein | MAG2890 | 75 ^d^ | Homologs in several mollicutes and non mollicute bacteria |
| MSC_0531 | triacylglycerol lipase | MAG0040 | 76 ^d^ | Homologs in several mollicutes |
| MSC_0517 | Glycerol ABC transporter, permease component | MAG6540 | 77 ^d^ | Homologs in several mollicutes |
| MSC_0118 | HEXOSEPHOSPHATE TRANSPORT PROTEIN | MAG4970 | 80 ^d^ | Homologs in several mollicutes and non mollicute bacteria |
| MSC_0801 | ABC transporter, permease component | MAG2710 | 89 ^d^ | Homologs in several mollicutes |
| MSC_0788 | Alkylphospho -te ABC transporter, permease component | MAG2710 | 89 ^d^ | Homologs in several mollicutes and non mollicute bacteria |
| MSC_0094 | Conserved HYPOTHETICAL PROTEIN | MAG5670 | 94 | Homologs in mollicutes and non mollicute bacteria |
| MSC_0093 | Conserved HYPOTHETICAL PROTEIN | MAG5660 | 94 | Homologs in several mollicutes and non mollicute bacteria |
| MSC_0621 | Conserved hypothetical protein | MAG2910 | 96 | Homologs in several mollicutes |
| MSC_0322 | conserved hypothetical prolipoprotein (abc transporter?) | MAG4620 | 96 | Homologs in several mollicutes |
| MSC_0636 | conserved hypothetical protein | MAG2860 | 97 | Homologs in several mollicutes |
| MSC_0626 | Conserved hypothetical transmembrane protein | MAG2850 | 97 | Homologs in several mollicutes |
| MSC_0925 | Conserved hypothetical transmembrane protein | MAG4600 | 97 | Homologs in several mollicutes |
| MSC_0897 | Prolipoprotein | MAG2960 | 97 | Homolog in the pneumoniae group |
| MSC_0320 | Conserved hypothetical transmembrane protein | MAG4640 | 98 | Homologs in several mollicutes |
| MSC_0637 | conserved hypothetical transmembrane protein | MAG2850 | 99 | Homologs in several mollicutes |
| MSC_0624 | Conserved hypothetical transmembrane protein | MAG2880 | 99 | Homologs in several mollicutes |
| MSC_0529 | Glycine cleavage system H protein | MAG0090 | 99 | Homologs in the Hominis group |
| MSC_0649 | EXODEOXYRIBONUCLEASE V ALPHA CHAIN | MAG2150 | 99 | Homologs in the Hominis group |
| MSC_0775 | Prolipoprotein | MAG4950 | 99 | Homologs in several mollicutes |
| MSC_0323 | ABC transporter, ATP-binding component ( -+?) | MAG4610 | 99 | Homologs in several mollicutes and non mollicute bacteria |
| MSC_0324 | ABC transporter, permease component ( -+?) | MAG4600 | 99 | Homologs in several mollicutes and non mollicute bacteria |
| MSC_0922 | hypothetical prolipoprotein | MAG4620 | 99 | Homologs in several mollicutes |
| MSC_0919 | Conserved hypothetical transmembrane protein | MAG4600 | 99 | Homologs in several mollicutes |
| MSC_0963 | Conserved hypothetical protein | MAG0390 | 100 | Homologs in several mollicutes |
| MSC_0964 | Oligopeptide ABC transporter, substrate-binding component | MAG0380 | 100 | Homologs in several mollicutes |
| MSC_0965 | oligopeptide ABC transporter, permease component | MAG0370 | 100 | Homologs in several mollicutes |
| MSC_0966 | oligopeptide ABC transporter, permease component | MAG0360 | 100 | Homologs in several mollicutes |
| MSC_0968 | oligopeptide ABC transporter, ATP-binding component | **MAG0340** | 100 | Homologs in several mollicutes and non mollicute bacteria |
| MSC_0967 | Oligopeptide ABC transporter, ATP-binding component | MAG0350 | 100 | Homologs in several mollicutes and non mollicute bacteria |
| MSC_0975 | oligopeptide ABC transporter, permease component | **MAG0340** | 100 | Homologs in several mollicutes |
| MSC_0983 | Oligopeptide ABC transporter, ATP-binding component | **MAG0340** | 100 | Homologs in several mollicutes |
| MSC_0257 | glycerol uptake facilitator | MAG4480 | 100 | Homologs in several mollicutes and non mollicute bacteria |
| MSC_0258 | glycerol ki -se | MAG4470 | 100 | Homologs in several mollicutes and non mollicute bacteria |
| MSC_0301 | oxidoreductase | MAG4120 | 100 | Homolog in the pneumoniae group |
| MSC_0295 | OXIDOREDUCTASE | MAG4120 | 100 | Homolog in the pneumoniae group |
| MSC_0631 | Conserved hypothetical protein | **MAG2840** | 100 | Homologs in several mollicutes |
| MSC_0627 | Prolipoprotein | **MAG2840** | 100 | Homologs in several mollicutes |
| MSC_0635 | Prolipoprotein | **MAG2840** | 100 | Homologs in several mollicutes |
| MSC_0625 | Prolipoprotein | MAG2870 | 100 | Homologs in several mollicutes |
| MSC_0628 | conserved hypothetical transmembrane protein | MAG2850 | 100 | Homologs in several mollicutes |
| MSC_0633 | conserved hypothetical transmembrane protein | MAG2850 | 100 | Homologs in several mollicutes |
| MSC_0622 | Conserved HYPOTHETICAL PROTEIN | MAG2900 | 100 | Homologs in several mollicutes and non mollicute bacteria |
| MSC_0620 | Conserved hypothetical transmembrane protein | MAG2920 | 100 | Homologs in several mollicutes |
| MSC_0619 | ATP synthase alpha chain | MAG2930 | 100 | Homologs in several mollicutes and non mollicute bacteria |
| MSC_0618 | ATP synthase beta chain | MAG2940 | 100 | Homologs in several mollicutes and non mollicute bacteria |
| MSC_0527 | lipoate-protein ligase A | MAG0070 | 100 | Homologs in several mollicutes |
| MSC_0526 | -DH dependent flavin oxidoreductase | MAG0050 | 100 | Homologs in several mollicutes |
| MSC_0519 | Prolipoprotein B | MAG6520 | 100 | Homologs in several mollicutes |
| MSC_0518 | Glycerol ABC transporter, permease component | MAG6530 | 100 | Homologs in several mollicutes |
| MSC_0696 | endopeptidase O | MAG3680 | 100 | Homologs in several mollicutes and non mollicute bacteria |
| MSC_0503 | 5-formyltetrahydrofolate cyclo-ligase | MAG5750 | 100 | Homologs in several mollicutes and non mollicute bacteria |
| MSC_0368 | triacylglycerol lipase | MAG1500 | 100 | Homologs in several mollicutes and non mollicute bacteria |
| MSC_0375 | glucose-inhibited division protein | MAG1470 | 100 | Homologs in several mollicutes and non mollicute bacteria |
| MSC_0774 | PEPTIDE METHIONINE SULFOXIDE REDUCTASE | MAG3130 | 100 | Homologs in several mollicutes and non mollicute bacteria |
| MSC_0778 | Conserved hypothetical protein | MAG3290 | 100 | Homologs in several mollicutes |
| MSC_0789 | alkylphospho -te ABC transporter, ATP-binding component | MAG2700 | 100 | Homologs in several mollicutes |
| MSC_0803 | alkylphospho -te ABC transporter, ATP-binding component | MAG2700 | 100 | Homologs in several mollicutes |
| MSC_0790 | Alkylphospho -te ABC transporter, substrate-bindin | **MAG2690** | 100 | Homologs in several mollicutes |
| MSC_0804 | ABC TRANSPORTER, substrate-binding component | **MAG2690** | 100 | Homologs in several mollicutes |
| MSC_0924 | Hypothetical prolipoprotein | MAG4640 | 100 | Homologs in several mollicutes |
| MSC_0903 | Hexose phosphate transport protein | MAG4970 | 100 | Homologs in several mollicutes |
| MSC_1015 | ASPARTATE-AMMONIA LIGASE | MAG0640 | 100 | Homologs in several mollicutes |
| MSC_1040 | Aspartate--ammonia ligase | MAG0640 | 100 | Homologs in several mollicutes |
| MSC_1019 | -DH oxidase | **MAG2460** | 100 | Homologs in several mollicutes and non mollicute bacteria |
| MSC_1044 | -DH oxidase | **MAG2460** | 100 | Homologs in several mollicutes and non mollicute bacteria |
| MSC_0233 | Conserved hypothetical protein | MAG5660 | - | Homolog in the pneumoniae group |
| MSC_0798 | Prolipoprotein | **MAG4950** | - | Homologs in the Hominis group |
| MSC_0782 | Prolipoprotein | **MAG4950** | - | Homologs in the Hominis group |
| MSC_0776 | Conserved hypothetical prolipoprotein | MAG4950 | - | Homologs in the Hominis group |
| MSC_0926 | ABC transporter, ATP-binding component | MAG4610 | - | Homolog in the pneumoniae group |
| MSC_0034 | D-LACTATE DEHYDROGE -SE | MAG1490 | - | Homolog in the pneumoniae group |
| MSC_0035 | MALATE PERMEASE | MAG4890 | - | Homolog in the pneumoniae group |
|  |  |  |  |  |
| MSC_0378 | conserved hypothetical protein | UU038 | 100 | Homolog in the pneumoniae group |
| MSC_0699 | transposase (IS1138-like) | MYPU_7560 | - | Homologs in several mollicutes |

^a^ Mnemonic the Best BLAST Hit (BBH) MAG: *M. agalactiae;* MGA: *M. gallisepticum* (shaded cells): mhp:*M. hyopneumoniae*; UU: *U. Urealyticum*; MYPU: *M. pulmonis*. Mnemonic in bold or red appears at least twice in the table.

^b^ bootstrap value supporting the association *M. mycoides subsp. mycoides SC* with its BBH in a phylogenetic reconstruction using the neighbour-joining method (bootstrap test with 500 replicates)

^c^ bootstrap test non applicable: no or too few other homolog; unclear phylogeny

^d^ Non significant bootstrap value
