# Supplementary material for: Being Pathogenic, Plastic, and Sexual while Living with a Nearly Minimal Bacterial Genome
Source: PLoS Genet. 2007 May 18;3(5):e75. doi: 10.1371/journal.pgen.0030075 (PMC1868952; doi:10.1371/journal.pgen.0030075)
Supplement: Supplementary file 13 [file pgen.0030075.st006.doc]

**Table S6**: **CDS candidates for HGT in between *M. capricolum subsp. capricolum* (MCAP) and *M. agalactiae* (MAG)**

| Mnemonic | Product | BBH^a^ | Bootstrap values^b^ (%) | Comment |
| --- | --- | --- | --- | --- |
| **MCAP CDS having homologs only in the Hominis group** | |  |  |  |
| MCAP0042 | lipoprotein, putative | MAG6040 | -^c^ | No other homolog |
| MCAP0198 | chromate ion transporter (CHR) family, putative | MAG6760 | 42^d^ | Several homologs in the hominis group |
| MCAP0440 | membrane protein, putative | MAG1640 | - | No other homolog |
| MCAP0442 | lipoprotein, putative | MAG1550 | - | No other homolog |
| MCAP0561 | lipoprotein, putative | MAG1550 | - | No other homolog |
| MCAP0564 | membrane protein, putative | MAG3930 | - | No other homolog |
| MCAP0566 | hypothetical protein | MAG3900 | - | No other homolog |
| MCAP0568 | hypothetical protein | MAG3870 | - | No other homolog |
| MCAP0571 | hypothetical protein | MAG3860 | - | Homolog in *M. hyopneumoniae* |
| MCAP0627 | membrane protein, putative | MAG1210 | - | Homolog in *M. pulmonis* |
|  |  |  |  |  |
| **MCAP CDS having an ortholog in the Spiroplasma group and an homolog only in *M. agalactiae*** | | | |  |
| MCAP0033 | lipoprotein, putative | **MAG1670** | - | No other homolog outside of the spiroplasma group |
| MCAP0034 | lipoprotein, putative (LppA family) | **MAG1670** | - | No other homolog outside of the spiroplasma group |
| MCAP0134 | membrane protein, putative | MAG3270 | - | No other homolog outside of the spiroplasma group, DUF285 family |
| MCAP0135 | membrane protein, putative | MAG4220 | - | No other homolog outside of the spiroplasma group, DUF285 family |
| MCAP0173 | hypothetical protein | MAG0230 | - | No other homolog outside of the spiroplasma group |
| MCAP0177 | conserved hypothetical protein | MAG1330 | - | No other homolog outside of the spiroplasma group |
| MCAP0188 | membrane protein, putative [C terminal extension of MCAP0166], interuption-C | MAG4220 | - | No other homolog outside of the spiroplasma group, DUF285 family |
| MCAP0211 | lipoprotein, putative | MAG1340 | - | No other homolog outside of the spiroplasma group, DUF285 family |
| MCAP0212 | lipoprotein, putative | MAG1330 | - | No other homolog outside of the spiroplasma group, DUF285 family |
| MCAP0240 | membrane protein, putative | MAG5760 | - | No other homolog outside of the spiroplasma group |
| MCAP0242 | lipoprotein, putative | MAG3270 | - | No other homolog outside of the spiroplasma group |
| MCAP0268 | lipoprotein, putative | **MAG2430** | - | No other homolog outside of the spiroplasma group |
| MCAP0269 | lipoprotein, putative | **MAG2430** | - | No other homolog outside of the spiroplasma group |
| MCAP0288 | conserved hypothetical protein | MAG3260 | - | No other homolog outside of the spiroplasma group, DUF285 family |
| MCAP0291 | conserved hypothetical protein | MAG4220 | - | No other homolog outside of the spiroplasma group, DUF285 family |
| MCAP0293 | conserved hypothetical protein | MAG1330 | - | No other homolog outside of the spiroplasma group, DUF285 family |
| MCAP0300 | conserved hypothetical protein | MAG1340 | - | No other homolog outside of the spiroplasma group, DUF285 family |
| MCAP0302 | conserved hypothetical protein | MAG1330 | - | No other homolog outside of the spiroplasma group, DUF285 family |
| MCAP0308 | conserved hypothetical protein | MAG1330 | - | No other homolog outside of the spiroplasma group, DUF285 family |
| MCAP0310 | conserved hypothetical protein | MAG1340 | - | No other homolog outside of the spiroplasma group, DUF285 family |
| MCAP0312 | lipoprotein, putative | MAG6480 | - | No other homolog outside of the spiroplasma group, DUF285 family |
| MCAP0328 | membrane protein, putative | MAG5760 | 89 ^d^ | No other homolog outside of the spiroplasma group |
| MCAP0329 | lipoprotein, putative | MAG5770 | 100 | No other homolog outside of the spiroplasma group |
| MCAP0444 | membrane protein, putative | MAG4220 | - | No other homolog outside of the spiroplasma group, DUF285 family |
| MCAP0448 | conserved hypothetical protein | MAG0080 | - | No other homolog outside of the spiroplasma group |
| MCAP0493 | hypothetical protein | MAG6440 | - | No other homolog outside of the spiroplasma group |
| MCAP0494 | hypothetical protein | MAG6450 | - | No other homolog outside of the spiroplasma group |
| MCAP0513 | lipoprotein, putative | **MAG6410** | - | No other homolog outside of the spiroplasma group |
| MCAP0514 | lipoprotein, putative | **MAG6410** | - | No other homolog outside of the spiroplasma group |
| MCAP0557 | membrane protein, putative | MAG6480 | - | No other homolog outside of the spiroplasma group, DUF285 family |
| MCAP0607 | lipoprotein, putative | MAG6410 | - | No other homolog outside of the spiroplasma group |
| MCAP0622 | membrane protein, putative | MAG3270 | - | No other homolog outside of the spiroplasma group, DUF285 family |
| MCAP0638 | membrane protein, putative | MAG6570 | - | No other homolog outside of the spiroplasma group |
| MCAP0720 | lipoprotein, putative | **MAG7270** | - | No other homolog outside of the spiroplasma group, DUF285 family |
| MCAP0721 | lipoprotein, putative | **MAG7270** | - | No other homolog outside of the spiroplasma group, DUF285 family |
| MCAP0728 | membrane protein, putative | MAG4940 | - | No other homolog outside of the spiroplasma group |
| MCAP0735 | membrane protein, putative | MAG3260 | - | No other homolog outside of the spiroplasma group, DUF285 family |
| MCAP0736 | membrane protein, putative | **MAG3270** | - | No other homolog outside of the spiroplasma group, DUF285 family |
| MCAP0737 | membrane protein, putative | **MAG3270** | - | No other homolog outside of the spiroplasma group, DUF285 family |
| MCAP0738 | membrane protein, putative | **MAG3270** | - | No other homolog outside of the spiroplasma group, DUF285 family |
| MCAP0758 | lipoprotein, putative | MAG6410 | - | No other homolog outside of the spiroplasma group |
| MCAP0763 | membrane protein, putative | MAG6480 | - | No other homolog outside of the spiroplasma group, DUF285 family |
| MCAP0770 | lipoprotein, putative | MAG2430 | - | No other homolog outside of the spiroplasma group, DUF285 family |
| MCAP0820 | conserved hypothetical protein | MAG2720 | - | No other homolog outside of the spiroplasma group |
| MCAP0843 | lipoprotein, putative | MAG6410 | - | No other homolog outside of the spiroplasma group |
| MCAP0846 | lipoprotein, putative | MAG4130 | - | No other homolog outside of the spiroplasma group |
| MCAP0857 | proton-glutamate symporter family protein | MAG2470 | 100 | No other homolog outside of the spiroplasma group |
|  |  |  |  |  |
| **MCAP CDS having orthologs in the Spiroplasma group and in several homologs in mollicutes** | | |  |  |
| MCAP0072 | lipoprotein, putative | MAG2960 | 91 | Homolog in the Pneumoniae group |
| MCAP0115 | membrane protein, putative | MAG0390 | 100 | Homologs in several mollicutes |
| MCAP0116 | oligopeptide ABC transporter, oligopeptide-binding protein, putative | MAG0380 | 100 | Homologs in several mollicutes |
| MCAP0117 | oligopeptide ABC transporter, permease protein | MAG0370 | 100 | Homologs in several mollicutes |
| MCAP0118 | oligopeptide ABC transporter, permease protein | MAG0360 | 100 | Homologs in several mollicutes |
| MCAP0119 | oligopeptide ABC transporter, ATP-binding protein | MAG0350 | 100 | Homologs in several mollicutes and non mollicute bacteria |
| MCAP0120 | oligopeptide ABC transporter, ATP-binding protein | MAG0340 | 100 | Homologs in several mollicutes and non mollicute bacteria |
| MCAP0196 | conserved hypothetical protein | MAG5660 | - | Homolog in the Pneumoniae group |
| MCAP0217 | glycerol uptake facilitator protein | MAG4480 | 100 | Homologs in several mollicutes and non mollicute bacteria |
| MCAP0218 | glycerol kinase | MAG4470 | 100 | Homologs in several mollicutes and non mollicute bacteria |
| MCAP0250 | Oxidoreductase, short chain dehydrogenase-reductase family | MAG4120 | 100 | Homolog in the Pneumoniae group |
| MCAP0256 | Oxidoreductase, short chain dehydrogenase-reductase family | MAG4120 | 100 | Homolog in the Pneumoniae group |
| MCAP0345 | membrane protein, putative | MAG2850 | 99 | Homologs in several mollicutes |
| MCAP0346 | lipoprotein, putative | **MAG2840** | 99 | Homologs in several mollicutes |
| MCAP0347 | membrane protein, putative | **MAG2850** | 100 | Homologs in several mollicutes |
| MCAP0348 | lipoprotein, putative | **MAG2840** | 100 | Homologs in several mollicutes |
| MCAP0349 | membrane protein, putative | **MAG2850** | 100 | Homologs in several mollicutes |
| MCAP0350 | lipoprotein, putative | **MAG2840** | 99 | Homologs in several mollicutes |
| MCAP0351 | membrane protein, putative | **MAG2830** | 100 | Homologs in several mollicutes |
| MCAP0352 | lipoprotein, putative | **MAG2840** | 99 | Homologs in several mollicutes |
| MCAP0353 | membrane protein, putative | MAG2880 | 99 | Homologs in several mollicutes |
| MCAP0354 | conserved hypothetical protein | MAG2890 | 75 ^d^ | Homologs in several mollicutes and non mollicute bacteria |
| MCAP0355 | conserved hypothetical protein | MAG2900 | 100 | Homologs in several mollicutes and non mollicute bacteria |
| MCAP0356 | conserved hypothetical protein | MAG2910 | 96 | Homologs in several mollicutes |
| MCAP0357 | membrane protein, putative | MAG2920 | 100 | Homologs in several mollicutes |
| MCAP0358 | ATP synthase F1, alpha subunit | MAG2930 | 100 | Homologs in several mollicutes and non mollicute bacteria |
| MCAP0359 | ATP synthase F1, beta subunit | MAG2940 | 100 | Homologs in several mollicutes and non mollicute bacteria |
| MCAP0445 | triacylglycerol lipase | MAG0040 | 76 ^d^ | Homologs in several mollicutes |
| MCAP0446 | triacylglycerol lipase, putative | MAG0030 | 68 ^d^ | Homologs in several mollicutes and non mollicute bacteria |
| MCAP0447 | glycine cleavage system H protein, putative | MAG0090 | 99 ^d^ | Several homologs in the Hominis group |
| MCAP0449 | lipoate-protein ligase | MAG0070 | 100 | Homologs in several mollicutes |
| MCAP0450 | NADH:flavin oxidoreductase - NADH oxidase family | MAG0050 | 100 | Homologs in several mollicutes |
| MCAP0451 | lipoprotein, putative | MAG6520 | 100 | Several homologs in the Hominis group |
| MCAP0452 | glycerol ABC transporter, permease | MAG6530 | 100 | Several homologs in the Hominis group |
| MCAP0453 | glycerol ABC transporter, permease | MAG6540 | 77 ^d^ | Several homologs in the Hominis group |
| MCAP0454 | glycerol ABC transporter, ATP-binding protein | MAG2310 | 100 | Several homologs in the Hominis group |
| MCAP0466 | endopeptidase O | MAG3680 | 100 | Homologs in several mollicutes and non mollicute bacteria |
| MCAP0467 | 5-formyltetrahydrofolate cyclo-ligase subfamily | MAG5750 | 100 | Homologs in several mollicutes and non mollicute bacteria |
| MCAP0559 | membrane protein, putative (TraG-TraD family) | MAG4040 | 100 | Homolog in the Hominis group |
| MCAP0565 | membrane protein, putative | MAG3910 | 100 | Homologs in the Hominis group |
| MCAP0567 | membrane protein, putative | MAG3890 | 100 | Hhomologs in the Hominis group |
| MCAP0606 | hydrolase, alpha-beta fold family | MAG1500 | 100 | Homologs in several mollicutes and non mollicute bacteria |
| MCAP0613 | glucose-inhibited division protein, putative | MAG1470 | 100 | Homologs in several mollicutes and non mollicute bacteria |
| MCAP0722 | peptide methionine sulfoxide reductase msrA-msrB | MAG3130 | 100 | Homologs in several mollicutes and non mollicute bacteria |
| MCAP0723 | lipoprotein, putative | MAG4950 | 99 | Several homologs in the Hominis group |
| MCAP0724 | membrane protein, putative | MAG4940 | - | Homologs in several mollicutes |
| MCAP0725 | conserved hypothetical protein | MAG3290 | 100 | Homologs in several mollicutes |
| MCAP0727 | lipoprotein, putative | MAG4950 | - | Homolog in the Hominis group |
| MCAP0729 | ABC transporter permease protein p69 | MAG2710 | 89 ^d^ | Several homologs in the Hominis group |
| MCAP0730 | ABC transporter, ATP-binding protein | MAG2700 | 100 | Homologs in several mollicutes |
| MCAP0731 | ABC transporter, substrate-binding protein | MAG2690 | 100 | Homologs in several mollicutes |
| MCAP0794 | membrane protein, putative | MAG4640 | 98 | Homologs in several mollicutes |
| MCAP0795 | membrane protein, putative | MAG4630 | 70 ^d^ | Several homologs in the Hominis group |
| MCAP0797 | ABC transporter, ATP-binding protein | MAG4610 | 99 | Homologs in several mollicutes and non mollicute bacteria |
| MCAP0798 | ABC transporter, permease protein, putative | MAG4600 | 99 | Homologs in several mollicutes and non mollicute bacteria |
| MCAP0799 | lipoprotein, putative | MAG4640 | 100 | Several homologs in the Hominis group |
| MCAP0800 | membrane protein, putative | MAG4630 | 70 | Several homologs in the Hominis group |
| MCAP0801 | lipoprotein, putative | MAG4620 | 99 | Homologs in several mollicutes |
| MCAP0802 | membrane protein, putative | MAG4600 | 99 | Homologs in several mollicutes |
| MCAP0814 | transporter protein, putative | MAG4970 | 100 | Homologs in several mollicutes |
| MCAP0817 | aspartate--ammonia ligase | MAG0640 | 100 | Homologs in several mollicutes |
| MCAP0857 | proton-glutamate symporter family protein | MAG2470 | 100 | No other homolog outside of the spiroplasma group |
| MCAP0858 | pyridine nucleotide-disulphide oxidoreductase | MAG2460 | 100 | Homologs in several mollicutes and non mollicute bacteria |
| MCAP0189 | multiple sugar ABC transporter, ATP-binding protein | MMOB3870 | 100 | Homologs in Phytoplasmas |
| **CDS having homologs only in the pneumoniae group** | |  |  |  |
| MCAP0048 | adenine-specific DNA methylase, putative | MYPE1780 | - | non mollicute bacteria |

^a^ Mnemonic of the Best BLAST Hit (BBH); MAG: *M. agalactiae;* MMOB: *M. mobile*; MYPR: *M. penetrans* Mnemonic in bold or in color appears at least twice in the table.

^b^ Bootstrap value supporting the association of *M. capricolum subsp. capricolum* with its homolog in a phylogenetic reconstruction using the neighbour-joining method (bootstrap test with 500 replicates)

^c^ Bootstrap test non applicable: no or too few other homolog; unclear phylogeny

^d^ Non significant bootstrap value
