# Supplementary material for: Being Pathogenic, Plastic, and Sexual while Living with a Nearly Minimal Bacterial Genome
Source: PLoS Genet. 2007 May 18;3(5):e75. doi: 10.1371/journal.pgen.0030075 (PMC1868952; doi:10.1371/journal.pgen.0030075)
Supplement: Supplementary file 14 [file pgen.0030075.st007.doc]

**Table S7** : **CDS candidates for HGT in between *M. penetrans* (PYPE) and other mollicutes**

| Mnemonic | Product | BBH^a^ | Bootstrap values^b^ (%) | Comment |
| --- | --- | --- | --- | --- |
| **MYPE CDS having no homolog in the pneuminiae group** | | |  |  |
| MYPE1730 | PTS system glucose-specific IIABC component | MYPU_3640 | -^c^ | Homologs in mollicutes and non mollicute bacteria |
| MYPE3320 | dihydroxyacetone kinase | MSC_0747 | - | Homologs in mollicutes and non mollicute bacteria |
| MYPE2650 | D-lactate dehydrogenase: D-LDH | MSC_0034 | - | Homologs in mollicutes |
| MYPE1240 | transposase for IS1630-like insertion sequence element | MYPU_3990 | - | Homolog in mollicutes and non mollicute bacteria |
| MYPE4880 | transposase for IS1630-like insertion sequence element | MYPU_3990 | - | Homolog in mollicutes and non mollicute bacteria |
| MYPE5350 | transposase for IS1630-like insertion sequence element | MYPU_3990 | - | Homolog in mollicutes and non mollicute bacteria |
| MYPE5400 | transposase for IS1630-like insertion sequence element | MYPU_3990 | - | Homolog in mollicutes and non mollicute bacteria |
| MYPE5660 | transposase for IS1630-like insertion sequence element | MYPU_3990 | - | Homolog in mollicutes and non mollicute bacteria |
| MYPE9780 | transposase for IS1630-like insertion sequence element | MYPU_3990 | - | Homolog in mollicutes and non mollicute bacteria |
| MYPE4550 | beta glucosidase | Mfl313 | - | Homolog in mollicutes |
| MYPE4560 | beta glucosidase | Mfl313 | - | Homolog in mollicutes |
| MYPE1780 | adenine-specific DNA methyltransferase | MCAP0048 | - | No other homolog |
| MYPE5820 | adenylosuccinate lyase | MCAP0766 | - | Homolog in mollicutes |
| MYPE2640 | permease | MSC_0035 | - | Homolog in mollicutes |
| MYPE3360 | diacylglycerol kinase | SPICI04_021 | - | No other homolog |
| MYPE1790 | adenine-specific DNA methyltransferase | MCAP0049 | - | Homologs in mollicutes |
| MYPE5220 | conserved hypothetical protein | MAG5660 | - | Homologs in mollicutes |
| MYPE1330 | lytB protein | SPICI03_258 | - | No other homolog |
| MYPE6340 | putative glycosyl transferase | MAG4860 | - | Homolog in mollicutes |
| MYPE3330 | dihydroxyacetone kinase | MSC_0276 | - | Homolog in mollicutes |
| MYPE1750 | N-acetylglucosamine 6-P deacetylase | MCAP0438 | - | Homologs in mollicutes |
| MYPE3200 | DNA-3-methyladenine glycosidase I | Mfl277 | - | No other homolog |
| MYPE3340 | PTS system enzyme I | MCAP0236 | - | Homologs in mollicutes |
| MYPE9650 | putative lipopolysaccharide biosynthesis enzyme KdtB | MSC_0272 | - | Homologs in mollicutes |
| MYPE6450 | conserved hypothetical protein | SPICI05_015 | - | No other homolog |
| MYPE7100 | transposase for IS150-like insertion sequence element | MSC_0904 | - | No other homolog |
| MYPE6430 | NADPH flavin oxidoreductase | MMOB3270 | - | Homologs in mollicutes |
| MYPE3070 | putative integral membrane protein | MMOB1970 | - | No other homolog |
| MYPE9460 | ribonuclease H-related protein | MSC_0328 | - | Homologs in mollicutes |
| MYPE4260 | 6-phosphogluconate dehydrogenase | MCAP0460 | - | No other homolog |
| MYPE9790 | hypothetical protein | MMOB6110 | - | No other homolog |
| MYPE7310 | conserved hypothetical | MSC_0036 | - | Homologs in mollicutes |
| MYPE280 | hypothetical protein | mhp555 | - | No other homolog |
| MYPE10210 | predicted transcriptional regulator | MS53_0006 | - | Homologs in mollicutes |
| MYPE1800 | conserved hypothetical protein | MCAP0050 | - | No other homolog |
| MYPE5720 | transposase for IS1202-like insertion sequence element | **SPICI04_042** | - | Homolog in mollicutes and non mollicute bacteria |
| MYPE170 | transposase | **SPICI04_042** | - | Homolog in mollicutes and non mollicute bacteria |
| MYPE5410 | transposase for IS1202-like insertion sequence element | **SPICI04_042** | - | Homolog in mollicutes and non mollicute bacteria |
| MYPE5710 | transposase for IS1202-like insertion sequence element | **SPICI04_042** | - | Homolog in mollicutes and non mollicute bacteria |
| MYPE7780 | transposase for IS1202-like insert sequence element | **SPICI04_042** | - | Homolog in mollicutes and non mollicute bacteria |
| MYPE2340 | transposase | **SPICI04_042** | - | Homolog in mollicutes and non mollicute bacteria |
| MYPE2310 | transposase for IS1202-like insertion sequence element | **SPICI04_042** | - | Homolog in mollicutes and non mollicute bacteria |
| MYPE3080 | ABC transporter ATP-binding protein | MMOB1960 | - | Homologs in mollicutes |
|  |  |  |  |  |
| **MYPE CDS having homolog in the pneuminiae group** | |  |  |  |
| MYPE6930 | amino acid permease | MS53_0423 | - | Homologs in mollicutes |

^a^ Mnemonic of the Best BLAST Hit (BBH) with MAG: *M. agalactiae;* MYPU: *M. pulmonis;* SPICI: *Spiroplasma citri*; mhp:*M. hyopneumoniae*; MCAP: *M. capricolum capricolum*; MSC: *M. mycoides mycoides SC*; MMOB: *M. mobile*; mfl: *Mesoplasma florum;* MS: *M. synoviae*. Mnemonic in bold or red appears at least twice in the table.

^b^ bootstrap value supporting the association *M. penetrans* with its homolog in a phylogenetic reconstruction using the neighbour-joining method (bootstrap test with 500 replicates)

^c^ bootstrap test non applicable: no or too few other homolog; unclear phylogeny
