# Supplementary material for: Being Pathogenic, Plastic, and Sexual while Living with a Nearly Minimal Bacterial Genome
Source: PLoS Genet. 2007 May 18;3(5):e75. doi: 10.1371/journal.pgen.0030075 (PMC1868952; doi:10.1371/journal.pgen.0030075)
Supplement: Supplementary file 15 [file pgen.0030075.st008.doc]

Table S8 : **CDS candidates for HGT in between *M. gallisepticum* (MGA) and *M. synoviae* (MS) or other mollicutes**

| Mnemonic | Product | BBH ^a^ | Bootstrap values^b^ (%) | Comment |
| --- | --- | --- | --- | --- |
| **MGA CDS having no homolog in the pneumoniae group** | |  |  |  |
| MGA_0965 | neutrophil activating factor | MS53_0300 | 39^d^ | Homologs in the Hominis group |
| MGA_1265 | conserved hypothetical | MS53_0107 | 60^d^ | Homologs in other mollicutes and non mollicutes bacteria |
| MGA_0798 | AprE-like | **MS53_0036** | 84^d^ | Homologs in the Hominis group |
| MGA_0801 | AprE-like | **MS53_0036** | 84^d^ | Homologs in the Hominis group |
| MGA_0815 | AprE-like | **MS53_0036** | 84^d^ | Homologs in the Hominis group |
| MGA_0906 | AprE-like | **MS53_0036** | 84^d^ | Homologs in the Hominis group |
| MGA_0090 | predicted phospholipid-binding protein | MS53_0539 | 97 | Homologs in the Hominis group |
| MGA_1091 | LepB | MS53_0040 | 100 | Homologs in the Hominis group |
| MGA_1088 | ABC transporter ATPase component | **MS53_0041** | 100 | Homologs in other mollicutes |
| MGA_1089 | ABC transporter ATP-binding protein | **MS53_0041** | 100 | Homologs in other mollicutes |
| MGA_1083 | conserved hypothetical | MS53_0044 | 100 | Homologs in the Hominis group |
| MGA_1109 | predicted transposase | MS53_0079 | 100 | Homologs in other mollicutes |
| MGA_1107 | conserved hypothetical | MS53_0172 | 100 | Homologs in other mollicutes |
| MGA_1102 | conserved hypothetical | MS53_0293 | 100 | Homologs in the Hominis group |
| MGA_1103 | conserved hypothetical | MS53_0294 | 100 | Homologs in the Hominis group |
| MGA_1087 | unique hypothetical | MS53_0042 | -^c^ | No other homolog |
| MGA_1085 | conserved hypothetical | MS53_0043 | - | No other homolog |
| MGA_1108 | unique hypothetical | MS53_0079 | - | Homologs in the Hominis group |
| MGA_0281 | Kef | MS53_0126 | - | No other homolog |
| MGA_0329 | conserved hypothetical | MS53_0199 | - | No other homolog |
| MGA_0076 | VlhA.1.06 | MS53_0229 | - | No other homolog |
| MGA_0984 | VlhA.4.10 | MS53_0241 | - | No other homolog |
| MGA_0967 | VlhA.4.02 | MS53_0249 | - | No other homolog |
| MGA_1147 | unique hypothetical | MS53_0283 | - | No other homolog |
| MGA_0584 | unique hypothetical | MS53_0462 | - | No other homolog |
| MGA_1079 | unique hypothetical | MS53_0474 | - | Homologs in the Hominis group |
| MGA_1305 | MaoC | MS53_0536 | - | No other homolog |
| MGA_0833 | ACH-1 | MS53_0537 | - | Homologs in non mollicutes bacteria |
| MGA_0519 | conserved hypothetical | MS53_0582 | - | Homolog in the Hominis group |
| MGA_0523 | conserved hypothetical | MS53_0583 | - | Homolog in the Hominis group |
| MGA_0525 | conserved hypothetical | MS53_0584 | - | No other homolog |
| MGA_0526 | unique hypothetical | MS53_0585 | - | No other homolog |
| MGA_1153 | unique hypothetical | MS53_0590 | - | Homologs in other mollicutes |
| MGA_0648 | conserved hypothetical lipoprotein | MS53_0602 | - | Homologs in other mollicutes |
| MGA_1140 | ABC-type multidrug transport system permease | MS53_0658 | - | No other homolog |
| MGA_0535 | unique hypothetical | MSC_1061 | - | No other homolog |
| MGA_0536 | SpoVK | MSC_1062 | - | No other homolog |
| MGA_0829 | conserved hypothetical lipoprotein | MYPU_3230 | - | Homologs in other mollicutes |
| MGA_0993 | conserved hypothetical lipoprotein | MYPU_3230 | - | Homologs in other mollicutes |
| MGA_1073 | conserved hypothetical lipoprotein | MYPU_3230 | - | Homologs in other mollicutes |
| MGA_1077 | ABC transporter permease protein | MYPU_4980 | 99 | Homologs in other mollicutes |
| MGA_1263 | beta-PGM | MYPU_6350 | - | Homologs in other mollicutes and non mollicutes bacteria |
| MGA_0517 | AprE | MYPU_6550 | 84 ^d^ | Homologs in the Hominis group |
| MGA_1115 | unique hypothetical | MYPU_6620 | - | No other homolog |
| MGA_1112 | unique hypothetical | MYPU_6630 | - | No other homolog |
| MGA_0274 | conserved hypothetical | mhp650 | - | Homologs in other mollicutes |
|  |  |  |  |  |
| **MGA CDS having homolog in the pneumoniae group** | |  |  |  |
| MGA_1138 | CcmA | MS53_0657 | 100 | Homologs in other mollicutes and non mollicutes bacteria |
| MGA_0091 | predicted phospholipid-binding protein | MS53_0538 | 83 ^d^ | Homologs in other mollicutes |
| MGA_0562 | conserved hypothetical | **MS53_0471** | 100 | Homologs in other mollicutes and non mollicutes bacteria |
| MGA_0564 | conserved hypothetical | **MS53_0471** | 100 | Homologs in other mollicutes and non mollicutes bacteria |
| MGA_1321d | AtpD | MS53_0464 | 100 | Homologs in other mollicutes and non mollicutes bacteria |
| MGA_0565 | conserved hypothetical | **MS53_0463** | 100 | Homologs in other mollicutes |
| MGA_0579 | conserved hypothetical | **MS53_0463** | 100 | Homologs in other mollicutes |
| MGA_0583 | conserved hypothetical | MS53_0462 | 100 | Homologs in other mollicutes and non mollicutes bacteria |
| MGA_0701 | FolA | MS53_0396 | 56 ^d^ | Homologs in other mollicutes |
| MGA_0175 | Dgk | MS53_0380 | 39 ^d^ | Homologs in other mollicutes and non mollicutes bacteria |
| MGA_0330 | Glyceraldehyde 3-P dehydroge -se | MS53_0270 | 100 | Homologs in other mollicutes |
| MGA_0234 | DppC/OppC | MS53_0185 | 100 | Homologs in other mollicutes |
| MGA_0235 | DppB/OppB | MS53_0184 | - | Homologs in other mollicutes and non mollicutes bacteria |
| MGA_1092 | FusA | MS53_0047 | 100 | Homologs in other mollicutes and non mollicutes bacteria |
| MGA_0230 | OppF | MSC_0968 | - | Homologs in other mollicutes and non mollicutes bacteria |
| MGA_1076 | ABC transporter ATP-binding protein | MYPU_4970 | - | Homologs in other mollicutes and non mollicutes bacteria |
| MGA_1078 | ABC transporter permease protein | MYPU_4990 | 42 ^d^ | Homologs in other mollicutes |
| MGA_0174 | Dgk | MAG0170 | 39 ^d^ | Homologs in other mollicutes and non mollicutes bacteria |
| MGA_0537 | HsdM | MAG5730 | 88 ^d^ | Homologs in other mollicutes |
| MGA_1100 | AsnS | mhp497 | - | Homologs in other mollicutes and non mollicutes bacteria |

^a^ Mnemonic of the Best BLAST Hit (BBH) MS: *M. synoviae*; MAG: *M. agalactiae;* MYPU: *M. pulmonis;* mhp: *M. hyopneumoniae*; MSC: *M. mycoides* subsp*. mycoides* SC;. Mnemonic in bold or red appears at least twice in the table..

^b^ bootstrap value supporting the association *M. penetrans* with its homolog in a phylogenetic reconstruction using the neighbour-joining method (bootstrap test with 500 replicates)

^c^ bootstrap test non applicable: no or too few other homolog; unclear phylogeny
