# Supplementary material for: Being Pathogenic, Plastic, and Sexual while Living with a Nearly Minimal Bacterial Genome
Source: PLoS Genet. 2007 May 18;3(5):e75. doi: 10.1371/journal.pgen.0030075 (PMC1868952; doi:10.1371/journal.pgen.0030075)
Supplement: Supplementary file 16 [file pgen.0030075.st009.doc]

**Table S9** : **CDS candidates for HGT in between *M. synoviae* (MS) and *M. galliepticum* (MGA)**

| Mnemonic | Product | BBH ^a^ | Bootstrap values^b^ (%) | Comment |
| --- | --- | --- | --- | --- |
| **CDS of MS having no homologs outside of the Pneumoniae group** | | |  |  |
| MS53_0042 | hypothetical protein | MGA_1087^b^ | -^c^ | No other homolog |
| MS53_0043 | hypothetical protein | MGA_1085 | - | No other homolog |
| MS53_0044 | hypothetical protein | **MGA_1083** | - | No other homolog |
| MS53_0045 | hypothetical protein | **MGA_1083** | - | No other homolog |
| MS53_0199 | hypothetical protein | MGA_0329 | - | No other homolog |
| MS53_0206 | putative phase-variable hemagglutinin | MGA_0984 | - | No other homolog |
| MS53_0207 | putative phase-variable hemagglutinin | MGA_0984 | - | No other homolog |
| MS53_0212 | putative phase-variable hemagglutinin | MGA_0984 | - | No other homolog |
| MS53_0213 | putative phase-variable hemagglutinin | MGA_0984 | - | No other homolog |
| MS53_0218 | putative phase-variable hemagglutinin | MGA_0984 | - | No other homolog |
| MS53_0231 | putative phase-variable hemagglutinin | MGA_0984 | - | No other homolog |
| MS53_0234 | putative phase-variable hemagglutinin | MGA_0984 | - | No other homolog |
| MS53_0236 | putative phase-variable hemagglutinin | MGA_0984 | - | No other homolog |
| MS53_0239 | putative phase-variable hemagglutinin | MGA_0984 | - | No other homolog |
| MS53_0245 | putative phase-variable hemagglutinin | MGA_0984 | - | No other homolog |
| MS53_0246 | putative phase-variable hemagglutinin | MGA_0984 | - | No other homolog |
| MS53_0255 | putative phase-variable hemagglutinin | MGA_0984 | - | No other homolog |
| MS53_0256 | putative phase-variable hemagglutinin | MGA_0984 | - | No other homolog |
| MS53_0265 | putative phase-variable hemagglutinin | MGA_0984 | - | No other homolog |
| MS53_0201 | putative phase-variable hemagglutinin | MGA_0967 | - | No other homolog |
| MS53_0202 | putative phase-variable hemagglutinin | MGA_0967 | - | No other homolog |
| MS53_0203 | putative phase-variable hemagglutinin | MGA_0967 | - | No other homolog |
| MS53_0204 | putative phase-variable hemagglutinin | MGA_0967 | - | No other homolog |
| MS53_0205 | putative phase-variable hemagglutinin | MGA_0967 | - | No other homolog |
| MS53_0209 | putative phase-variable hemagglutinin | MGA_0967 | - | No other homolog |
| MS53_0210 | putative phase-variable hemagglutinin | MGA_0967 | - | No other homolog |
| MS53_0217 | putative phase-variable hemagglutinin | MGA_0967 | - | No other homolog |
| MS53_0221 | putative phase-variable hemagglutinin | MGA_0967 | - | No other homolog |
| MS53_0222 | putative phase-variable hemagglutinin | MGA_0967 | - | No other homolog |
| MS53_0223 | putative phase-variable hemagglutinin | MGA_0967 | - | No other homolog |
| MS53_0224 | putative phase-variable hemagglutinin | MGA_0967 | - | No other homolog |
| MS53_0225 | putative phase-variable hemagglutinin | MGA_0967 | - | No other homolog |
| MS53_0226 | putative phase-variable hemagglutinin | MGA_0967 | - | No other homolog |
| MS53_0228 | putative phase-variable hemagglutinin | MGA_0967 | - | No other homolog |
| MS53_0230 | putative phase-variable hemagglutinin | MGA_0967 | - | No other homolog |
| MS53_0232 | putative phase-variable hemagglutinin | MGA_0967 | - | No other homolog |
| MS53_0237 | putative phase-variable hemagglutinin | MGA_0967 | - | No other homolog |
| MS53_0238 | putative phase-variable hemagglutinin | MGA_0967 | - | No other homolog |
| MS53_0240 | putative phase-variable hemagglutinin | MGA_0967 | - | No other homolog |
| MS53_0241 | putative phase-variable hemagglutinin | MGA_0967 | - | No other homolog |
| MS53_0242 | putative phase-variable hemagglutinin | MGA_0967 | - | No other homolog |
| MS53_0243 | putative phase-variable hemagglutinin | MGA_0967 | - | No other homolog |
| MS53_0244 | putative phase-variable hemagglutinin | MGA_0967 | - | No other homolog |
| MS53_0247 | putative phase-variable hemagglutinin | MGA_0967 | - | No other homolog |
| MS53_0248 | putative phase-variable hemagglutinin | MGA_0967 | - | No other homolog |
| MS53_0249 | putative phase-variable hemagglutinin | MGA_0967 | - | No other homolog |
| MS53_0250 | putative phase-variable hemagglutinin | MGA_0967 | - | No other homolog |
| MS53_0252 | putative phase-variable hemagglutinin | MGA_0967 | - | No other homolog |
| MS53_0253 | putative phase-variable hemagglutinin | MGA_0967 | - | No other homolog |
| MS53_0257 | putative phase-variable hemagglutinin | MGA_0967 | - | No other homolog |
| MS53_0258 | putative phase-variable hemagglutinin | MGA_0967 | - | No other homolog |
| MS53_0259 | putative phase-variable hemagglutinin | MGA_0967 | - | No other homolog |
| MS53_0260 | putative phase-variable hemagglutinin | MGA_0967 | - | No other homolog |
| MS53_0261 | putative phase-variable hemagglutinin | MGA_0967 | - | No other homolog |
| MS53_0263 | putative phase-variable hemagglutinin | MGA_0967 | - | No other homolog |
| MS53_0266 | putative phase-variable hemagglutinin | MGA_0967 | - | No other homolog |
| MS53_0267 | putative phase-variable hemagglutinin | MGA_0967 | - | No other homolog |
| MS53_0268 | phase-variable hemagglutinin | MGA_0967 | - | No other homolog |
| MS53_0208 | putative phase-variable hemagglutinin | **MGA_0076** | - | No other homolog |
| MS53_0211 | putative phase-variable hemagglutinin | **MGA_0076** | - | No other homolog |
| MS53_0214 | putative phase-variable hemagglutinin | **MGA_0076** | - | No other homolog |
| MS53_0215 | putative phase-variable hemagglutinin | **MGA_0076** | - | No other homolog |
| MS53_0216 | putative phase-variable hemagglutinin | **MGA_0076** | - | No other homolog |
| MS53_0219 | putative phase-variable hemagglutinin | **MGA_0076** | - | No other homolog |
| MS53_0220 | putative phase-variable hemagglutinin | **MGA_0076** | - | No other homolog |
| MS53_0227 | putative phase-variable hemagglutinin | **MGA_0076** | - | No other homolog |
| MS53_0229 | putative phase-variable hemagglutinin | **MGA_0076** | - | No other homolog |
| MS53_0233 | putative phase-variable hemagglutinin | **MGA_0076** | - | No other homolog |
| MS53_0235 | putative phase-variable hemagglutinin | **MGA_0076** | - | No other homolog |
| MS53_0251 | putative phase-variable hemagglutinin | **MGA_0076** | - | No other homolog |
| MS53_0254 | putative phase-variable hemagglutinin | **MGA_0076** | - | No other homolog |
| MS53_0262 | putative phase-variable hemagglutinin | **MGA_0076** | - | No other homolog |
| MS53_0264 | putative phase-variable hemagglutinin | **MGA_0076** | - | No other homolog |
| MS53_0269 | putative phase-variable hemagglutinin | **MGA_0076** | - | No other homolog |
| MS53_0290 | hypothetical protein | MGA_1100 | - | No other homolog |
| MS53_0443 | hypothetical protein | MGA_1103 | - | No other homolog |
| MS53_0474 | hypothetical protein | MGA_1079 | - | No other homolog |
| MS53_0476 | hypothetical protein | MGA_1108 | - | No other homolog |
| MS53_0490 | hypothetical protein | MGA_1271 | - | No other homolog |
| MS53_0536 | putative transcription regulatory protein | MGA_1305 | - | No other homolog |
| MS53_0537 | putative coenzyme A transferase | MGA_0833 | - | No other homolog |
| MS53_0584 | hypothetical protein | MGA_0525 | - | No other homolog |
| MS53_0589 | hypothetical protein | MGA_1147 | - | No other homolog |
| MS53_0590 | hypothetical protein | MGA_1153 | - | No other homolog |
| MS53_0658 | putative ABC transporter | MGA_1140 | - | No other homolog |
|  |  |  |  |  |
| **MS having ortholog in the Hominis group** | |  |  |  |
| MS53_0040 | signal peptidase I | MGA_1091 | 100 | No other homolog outside of the hominis group |
| MS53_0398 | thymidylate synthase | MGA_0699 | 83^d^ | Orthologs in several mollicutes |
| MS53_0039 | hypothetical protein | MGA_1092 | 99 | Orthologs in all other mollicutes and other bacteria |
| MS53_0036 | hypothetical protein | MGA_0815 | 98 | No other homolog outside of the hominis group |
| MS53_0041 | putative ABC transporter ATP-binding protein | MGA_1089 | 100 | No other homolog outside of the hominis group |
| MS53_0172 | hypothetical protein | MGA_1107 | 100 | Othologs in the spiroplasma group |
| MS53_0397 | dihydrofolate reductase | MGA_0701 | 73^d^ | No other homolog outside of the hominis group |
| MS53_0399 | ribonucleoside-diphosphate reductase beta chain | MGA_0698 | 100 | Orthologs in several mollicutes and non mollicutes bacteria |
| MS53_0400 | NrdI protein | MGA_0696 | 100 | Orthologs in several mollicutes and non mollicutes bacteria |
| MS53_0582 | hypothetical protein | MGA_0519 | - | No other homolog outside of the hominis group |
| MS53_0583 | hypothetical protein | **MGA_0523** | - | No other homolog outside of the hominis group |
| MS53_0586 | hypothetical protein | **MGA_0523** | - | No other homolog outside of the hominis group |
| MS53_0538 | hypothetical protein | MGA_0091 | 98 | No other homolog outside of the hominis group |
| MS53_0539 | hypothetical protein | MGA_0090 | 97 | No other homolog outside of the hominis group |
| MS53_0069 | hypothetical protein | **MGA_0583** | 99 | Orthologs in *U. urealyticum* |
| MS53_0070 | hypothetical protein | **MGA_0583** | 99 | Orthologs in *U. urealyticum* |
| MS53_0462 | hypothetical protein | **MGA_0583** | 99 | Orthologs in *U. urealyticum* |
| MS53_0471 | hypothetical protein | **MGA_0583** | 99 | Orthologs in *U. urealyticum* |
| MS53_0292 | hypothetical protein | MGA_1100 | 100 | No other homolog outside of the hominis group |
| MS53_0294 | hypothetical protein | **MGA_1103** | 100 | No other homolog outside of the hominis group |
| MS53_0442 | hypothetical protein | **MGA_1103** | 93 | No other homolog outside of the hominis group |
| MS53_0079 | putative transposase | MGA_1109 | 100 | No other homolog outside of the hominis group |
| MS53_0177 | putative transposase ISMHp1-like | MGA_1109 | 100 | No other homolog outside of the hominis group |
| MS53_0178 | putative transposase ISMHp1-like | MGA_1110 | 100 | No other homolog outside of the hominis group |
| MS53_0291 | hypothetical protein | MGA_1100 | - | No other homolog outside of the hominis group |
| MS53_0475 | putative transposase,ISMHp1-like | MGA_1109 | 100 | No other homolog outside of the hominis group |
| MS53_0657 | putative ABC transporter ATP-binding protein | MGA_1138 | 100 | Orthologs in mollicutes of the Pneumoniae and Spiroplasma groups |
| MS53_0293 | hypothetical protein | MGA_1102 | 100 | No other homolog outside of the hominis group |
| MS53_0463 | hypothetical protein | MGA_0565 | 100 | Orthologs in mollicutes of the Pneumoniae and Spiroplasma groups |
| MS53_0198 | acylneuraminate lyase | *M. alligatoris* AAR98793 | 100 | Orthologs in several mollicutes and non mollicutes bacteria |

^a^ Mnemonic of the Best BLAST Hit (BBH). MGA: *M. gallisepticum*. Mnemonic in bold or red appears at least twice in the table.

^b^ bootstrap value supporting the association *M. mycoides subsp. mycoides SC* with its homolog in a phylogenetic reconstruction using the neighbour-joining method (bootstrap test with 500 replicates)

^c^ bootstrap test non applicable: no or too few other homolog; unclear phylogeny

^d^ Non significant bootstrap value
