# Supplementary material for: Being Pathogenic, Plastic, and Sexual while Living with a Nearly Minimal Bacterial Genome
Source: PLoS Genet. 2007 May 18;3(5):e75. doi: 10.1371/journal.pgen.0030075 (PMC1868952; doi:10.1371/journal.pgen.0030075)
Supplement: Supplementary file 17 [file pgen.0030075.st010.doc]

**Table S10** : List of the 30 shared proteins (COGs;[1]) used for supertree construction

| Orthologous group | Annotation |
| --- | --- |
| COG0012 | Predicted GTPase, probable translation factor |
| COG0016 | Phenylalanine-tRNA synthethase alpha subunit |
| COG0048 | Ribosomal protein S12 |
| COG0049 | Ribosomal protein S7 |
| COG0052 | Ribosomal protein S2 |
| COG0080 | Ribosomal protein L11 |
| COG0081 | Ribosomal protein L1 |
| COG0087 | Ribosomal protein L3 |
| COG0091 | Ribosomal protein L2 |
| COG0092 | Ribosomal protein S3 |
| COG0093 | Ribosomal protein L14 |
| COG0094 | Ribosomal protein L5 |
| COG0096 | Ribosomal protein S8 |
| COG0097 | Ribosomal protein L6P/L9E |
| COG0098 | Ribosomal protein S5 |
| COG0099 | Ribosomal protein S13 |
| COG0100 | Ribosomal protein S11 |
| COG0102 | Ribosomal protein L13 |
| COG0103 | Ribosomal protein S9 |
| COG0172 | Seryl-tRNA synthetase |
| COG0184 | Ribosomal protein S15P/S13E |
| COG0186 | Ribosomal protein S17 |
| COG0197 | Ribosomal protein L16/L10E |
| COG0200 | Ribosomal protein L15 |
| COG0201 | Preprotein translocase subunit SecY |
| COG0202 | DNA-directed RNA polymerase, alpha subunit |
| COG0256 | Ribosomal protein L18 |
| COG0495 | Leucyl-tRNA synthetase |
| COG0522 | Ribosomal protein S4 and related proteins |
| COG0533 | Metal-dependent proteases with chaperone activity |

1. Tatusov RL, Koonin EV, Lipman DJ (1997) A genomic perspective on protein families. Science 278: 631-637.
